# Identification of the Immune Subtype of Hepatocellular Carcinoma for the Prediction of Disease-Free Survival Time and Prevention of Recurrence by Integrated Analysis of Bulk- and Single-Cell RNA Sequencing Data

**DOI:** 10.3389/fimmu.2022.868325

**Published:** 2022-06-06

**Authors:** Jie Fu, Xiaohua Lei

**Affiliations:** ^1^ Department of General Surgery, The Second Xiangya Hospital of Central South University, Changsha, China; ^2^ The First Affiliated Hospital, Department of Hepato-Biliary-Pancreatic Surgery, Hengyang Medical School, University of South China, Hengyang, China

**Keywords:** immune subtype, bulksequencing, single-cell, hepatocellular carcinoma, disease-free survival time, prevention of recurrence

## Abstract

**Background:**

The main factors affecting the long-term prognosis of hepatocellular carcinoma (HCC) patients undergoing radical surgery are recurrence and metastasis. However, the methods for predicting disease-free survival (DFS) time and preventing postoperative recurrence of HCC are still very limited.

**Methods:**

In this study, immune cell abundances in HCC samples were analyzed by single-sample gene set enrichment analysis (ssGSEA), while the prognostic values of immune cells for DFS time prediction were evaluated by the least absolute shrinkage and selection operator (LASSO) and subsequent univariate and multivariate Cox analyses. Next, a risk score was constructed based on the most prognostic immune cells and their corresponding coefficients. Interactions among prognostic immune cells and the specific targets for the prevention of recurrence were further identified by single-cell RNA (scRNA) sequencing data and CellMiner.

**Results:**

A novel efficient T cell risk score (TCRS) was constructed based on data from the three most prognostic immune cell types (effector memory CD8 T cells, regulatory T cells and follicular helper T cells) for identifying an immune subtype of HCC patients with longer DFS times and inflammatory immune characteristics. Functional differences between the high- and low-score groups separated by TCRS were clarified, and the cell-cell communication among these immune cells was elucidated. Finally, fifteen hub genes that may be potential therapeutic targets for the prevention of recurrence were identified.

**Conclusions:**

We constructed and verified a useful model for the prediction of DFS time of HCC after surgery. In addition, fifteen hub genes were identified as candidates for the prevention of recurrence, and a preliminarily investigation of potential drugs targeting these hub genes was carried out.

## Introduction

Liver cancer is the third leading cause of cancer-related death for both sexes and the second leading cause of cancer-related death for males worldwide (1). The most common type of liver cancer is hepatocellular carcinoma (HCC) (1). Great progress has been made in the treatments of HCC in recent years, but the prognosis is still unsatisfactory due to challenges including the lack of availability of early-diagnostic markers, early recurrence after radical surgery and resistance to chemotherapy or molecular-targeted therapy (2–6). Since the therapeutic efficacy of existing treatments for HCC patients in advanced stages of disease is very limited, it is particularly important to identify sensitive early diagnostic markers and predictors of disease-free survival (DFS) time, as well as reliable targets for the prevention of recurrence, which will greatly improve the DFS time and quality of life of HCC patients.

In recent years, some research has been carried out on the prediction of HCC recurrence after radical surgery or liver transplantation (7–11). For example, clinicopathological features such as spleen stiffness measurement (SSM) and the Metavir score were related to late recurrence of HCC (12), while albumin-bilirubin (ALBI) grade and high serum alpha-fetoprotein (AFP) were associated with early recurrence (13). In addition, some new technologies, such as radiomics and nanotechnology, have also been gradually applied to the prediction of DFS time and control of HCC recurrence (14–17). Moreover, a series of gene-based prognostic models were also constructed for the prediction of HCC recurrence (18, 19). However, the specific mechanisms for HCC recurrence remain unclear, and there is a lack of specific targets for preventing the recurrence of HCC.

The effect of immune characteristics on prognosis prediction and therapeutic response has been clarified in a variety of cancers (20–22). A recent study has also shown that tumor immune archetypes can be distinguished according to the comprehensive expression state of immune cells, which will help to judge the prognosis and choose the appropriate treatment scheme (23). Immunotherapy has become the recommended therapy for many diseases, including HCC (21, 24). In addition, immune characteristic differences between primary and early-relapsed HCC have recently been systemically investigated by integrated analysis of multiomics data (25). However, there is a lack of an immune-related risk score for the prediction of DFS time and efficient immune-related targets for the prevention of HCC recurrence.

In this study, a T cell risk score (TCRS) was developed based on bulk-sequencing data, and the prognostic value of this model was validated in a large number of HCC samples from different cohorts. This model was used to identify an immune subtype of HCC patients with longer DFS times and inflammatory immune characteristics. Moreover, hub genes that might associated with HCC recurrence were identified by single-cell RNA (scRNA) sequencing data. Finally, potential therapeutic drugs for preventing recurrence were preliminarily screened by CellMiner.

## Materials and Methods

### Data Acquisition and Preprocessing

Bulk-sequencing data in count or fragments per kilobase million (FPKM) forms and the survival information of 371 primary HCC patients were downloaded from The Cancer Genome Atlas (TCGA) database as a training cohort (https://portal.gdc.cancer.gov/). Next, the FPKM data and survival information of 159 patients from Zhongshan Hospital of Fudan University were downloaded from the NODE (https://www.biosino.org/node) database as validation cohort 1 (26), while the corresponding data of 242 patients from GSE14520 were downloaded from the Gene Expression Omnibus (GEO) (https://www.ncbi.nlm.nih.gov/geo/) database as validation cohort 2 (27). FPKM values were transformed into transcripts per million (TPM) values before analysis. If the patient relapses during postoperative follow-up, its status is defined as “Recurred”, otherwise it is defined as “Disease Free”. The time from operation to disease recurrence or the last follow-up was defined as DFS time. After preprocessing, a total of 672 HCC patients with DFS times ≥ 1 month (297 patients from the training cohort, 155 patients from validation cohort 1, and 220 patients from validation cohort 2) were analyzed in this study.

### Construction and Evaluation of the TCRS

The immune score, ESTIMATE score, stromal score and tumor purity of HCC samples were calculated by the “estimate” package in R (28), while the abundances of 28 immune cells in HCC samples were calculated by the single-sample gene set enrichment analysis (ssGSEA) method. In detail, expression data of tumor samples were used to calculate immune cell abundances according to gene set variation analysis (GSVA) algorithm and specific cell markers (**Supplementary Table 1**) as previously reported (29, 30). Next, prognostic values of these 28 immune cells for DFS prediction were evaluated by least absolute shrinkage and selection operator (LASSO) and subsequent univariate and multivariate Cox analyses. As a result, a TCRS was constructed based on the most prognostic immune cells (P value < 0.05) (effector memory CD8 T cells, regulatory T cells and follicular helper T cells) and their corresponding coefficients from multivariate Cox analysis. The formula is as follows: ∑ (abundance * coef). Abundance: abundances of prognostic immune cells, coef: risk coefficients of prognostic immune cells. HCC patients in each cohort were separated into high- and low-score groups according to the optimal cutoff value of the TCRS calculated by the “roc” method in the “ggrisk” package, and the survival and immune status between the two groups were compared.

### GSVA and Differential Analysis

In the training cohort, differences between the high- and low-score groups were preliminarily identified by GSVA (31). Next, differentially expressed genes (DEGs) between the high- and low-score groups were identified by the “DESeq2” package according to the count data (32). Subsequently, Gene Ontology (GO) and Kyoto Encyclopedia of Genes and Genomes (KEGG) analyses were performed by the “clusterProfiler” package using the significant DEGs (P < 0.05 and |log2FoldChange| > 1) (33).

### Analysis of scRNA Sequencing Data of HCC Patients

ScRNA sequencing data of twelve primary HCC samples (P08, P09, P10, P11, P12, P13, P14, P15, P16, P17, P18 and P19) and six early-relapsed HCC samples (P01, P02, P03, P04, P05 and P07) were downloaded from the China National GeneBank DataBase (CNSA: CNP0000650) and analyzed by the “Seurat” package (25, 34). Five cell clusters (C0, C1, C3, C5 and C19), including 5415 cells from primary HCC and 1879 cells from relapsed HCC were annotated with T cells by the data provider. The expression data of these T cells were analyzed in this study. Next, data normalization and identification of the highly variable genes (HVGs) were conducted by the “SCTransform” method. After principal component analysis (PCA), the 23 most powerful principal components (PCs) were used for uniform manifold approximation and projection (UMAP) analysis for dimension reduction. After that, fifteen T cell clusters were identified through the “FindNeighbors” and “FindClusters” functions. Compared with all other clusters, significant DEGs in each cluster were identified by the “FindAllMarkers” function. Subsequently, cell types were annotated by the specific markers as previously described (**Supplementary Table 2**) (35–37). Cell-cell communication among the cell types was evaluated by the “CellChat” package (38). In detail, gene expression data of annotated cells are used as input information, and the interaction of ligands, receptors and their cofactors are combined to simulate intercellular communication. This process is calculated through the built-in function of “CellChat”. In this process, ligands are defined as outgoing signals and receptors are defined as incoming signals. DEGs in the same type of immune cells between primary HCC cells and relapsed HCC cells were identified by the “FindMarkers” method.

### Evaluation of the Prognostic and Immune Values of the DEGs Identified by scRNA Sequencing Data

The prognostic values of the DEGs between immune cells were analyzed by univariate and multivariate Cox analyses, and the most prognostic genes (P < 0.05) were further screened by LASSO. As a result, a gene risk score (GRS) was calculated based on the remaining prognostic genes and their corresponding coefficients from multivariate Cox analysis. The formula is as follows: ∑ (exp * coef). Exp: expression of prognostic genes, coef: risk coefficients of prognostic genes. HCC patients in the training cohort were also separated into high- and low-score groups according to the optimal cutoff value of GRS calculated by the “roc” method in the “ggrisk” package, and the survival and immune status between the two groups were compared. Finally, the potential drugs targeting these genes for recurrence prevention were screened preliminarily by CellMiner, a web tool based on the NCI-60 cell line set (39).

### Statistical Analysis

In this study, all of the data were analyzed by R software (4.1.0). The survival data between different groups were compared by log-rank test. The continuous variables between two groups were compared by Wilcoxon test. Correlation analysis was performed by Pearson method. All statistical tests were two-sided, and p value < 0.05 was considered statistically significant.

## Results

### Construction and Evaluation of TCRS

**Figure 1:**
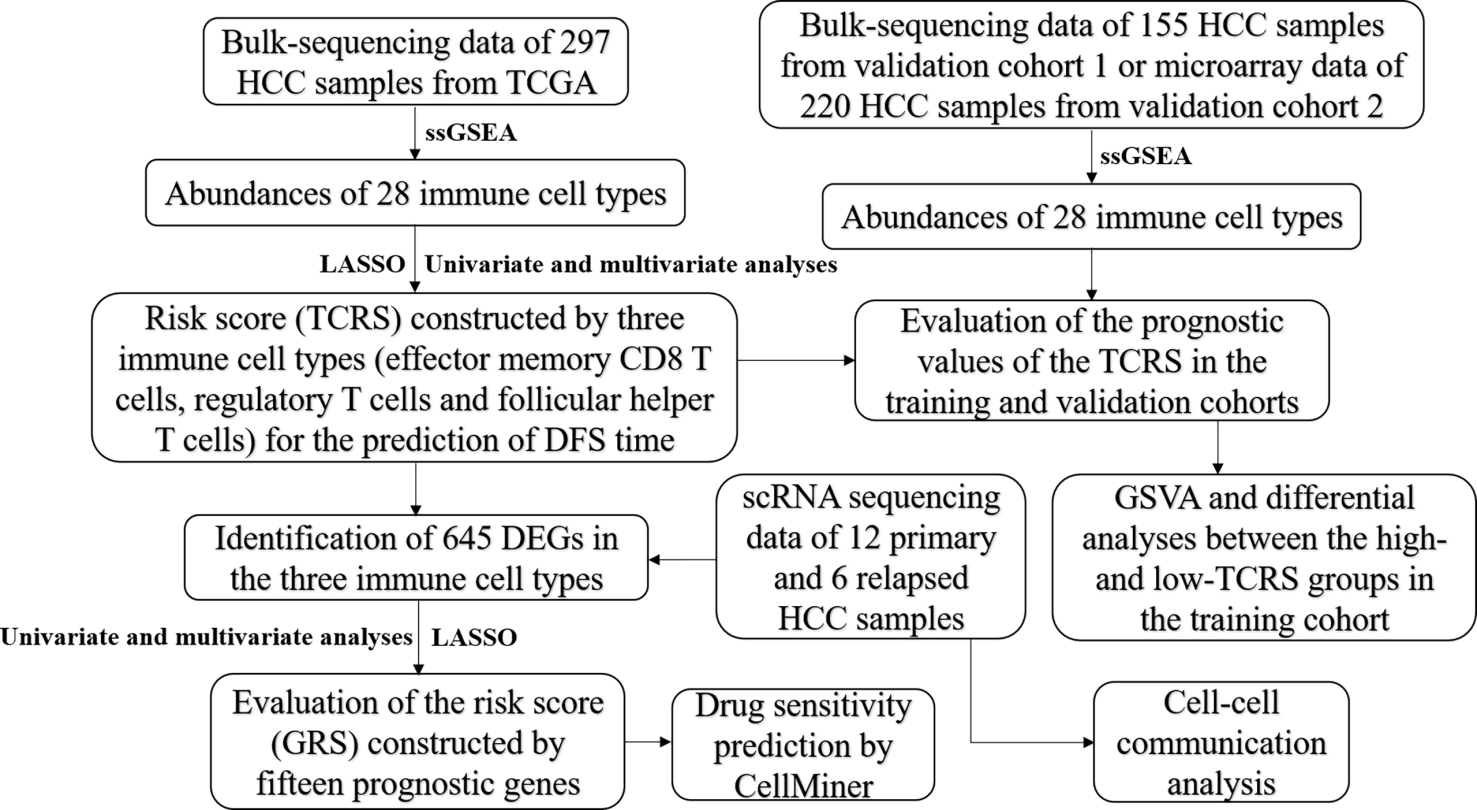
Schematic diagram of the analysis flow of this study.

**Figure 2:**
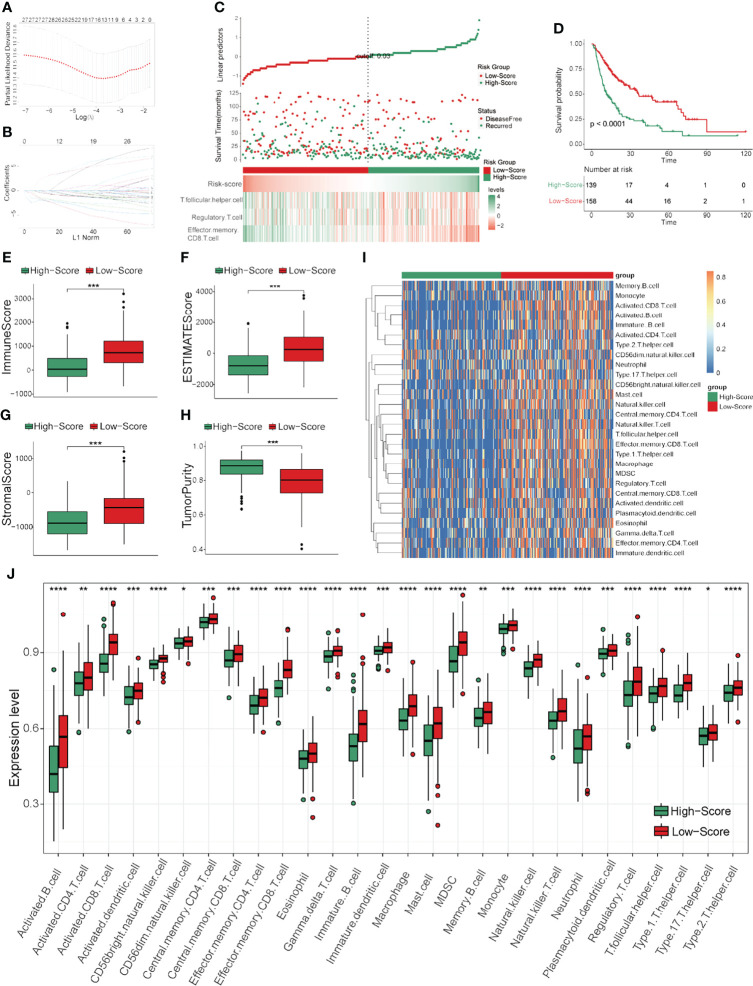
Construction and evaluation of the TCRS in the training cohort. **(A, B)** Cvfit and fit plots of LASSO screen. **(C)** Survival status and abundances of effector memory CD8 T cells, regulatory T cells and follicular helper T cells between the high- and low-score groups. **(D)** Kaplan-Meier curve for the two groups in the training cohort. **(E–H)** The expression levels of the immune score **(E)**, ESTIMATE score **(F)**, stromal score **(G)** and tumor purity **(H)** between the two groups. **(I, J)** The expression levels of 28 immune cells for the two groups visualized by heatmap **(I)** or boxplot **(J)**. *p < 0.05, **p < 0.01, ***p < 0.001, ****p < 0.0001.

The workflow of this study is shown in **Figure 1**. After screening by LASSO and subsequent univariate and multivariate Cox analyses (**Figures 2A, B** and **Table 1**), a TCRS was calculated based on the following formula: the abundance of effector memory CD8 T cells × (-13.088227) + the abundance of regulatory T cells × (2.831218) + the abundance of follicular helper T cells × (6.116813). The group information, DFS status and abundances of effector memory CD8 T cells, regulatory T cells and follicular helper T cells between the high- and low-score groups are shown in **Figure 2C**. The results of survival analyses showed that patients in the low-score group had a significantly longer DFS time than those in the high-score group (**Figure 2C, D**), and the area under receiver operating characteristic (ROC) curves (AUCs) for 1-, 3-, and 5-years DFS time prediction of the TCRS were 0.72, 0.64, and 0.7, respectively (**Supplementary Figure 1A**). In addition, the DFS time of disease-free patients was significantly longer than that of recurrent patients (**Supplementary Figure 2A**). Next, immune characteristics between the two groups were identified. We note that the immune score, ESTIMATE score and stromal score were significantly higher in the low-score group (**Figure 2E–G**), while tumor purity was significantly higher in the high-score group (**Figure 2H**). In addition, it is noteworthy that the expression levels of 28 immune cells were significantly higher in the low-score group (**Figure 2I, J**), which can be identified as an immune subtype of HCC with longer DFS time and inflammatory immune characteristics.

**Table 1:** Univariate and multivariate Cox regression analyses of the thirteen immune cell types.

Cell type	Univariate analysis			Multivariate analysis		
	HR	95% CI	P value	HR	95% CI	P value
Activated B cell	0.14	0.05-0.4	0	0.4	0.05-3.22	0.393
Activated CD4 T cell	1.5	0.23-9.82	0.675	NA	NA	NA
Activated CD8 T cell	0.01	0-0.11	0	0.12	0-13.34	0.38
CD56 bright natural killer cell	0	0-0	0	0.03	0-133.52	0.405
Effector memory CD8 T cell	0	0-0.04	0	0	0-0.7	0.039*
Eosinophil	0.03	0-0.37	0.006	0.06	0-1.43	0.082
Memory B cell	0.27	0.02-3.33	0.307	NA	NA	NA
Monocyte	0	0-0.14	0.006	0.14	0-136.41	0.575
Natural killer cell	0	0-0.1	0.001	1.07	0-383.76	0.983
Plasmacytoid dendritic cell	0.13	0-18.16	0.421	NA	NA	NA
Regulatory T cell	0.21	0.04-1.25	0.086	101.78	3.24-3193.81	0.009**
Follicular helper T cell	0.05	0-1.06	0.054	18498.04	23.05-1.48E+7	0.004**
Type 1 T helper cell	0	0-0.03	0	0	0-28.32	0.213

*p < 0.05, **p < 0.05.

NA, not available.

### External Validation of the TCRS Efficiency

**Figure 3:**
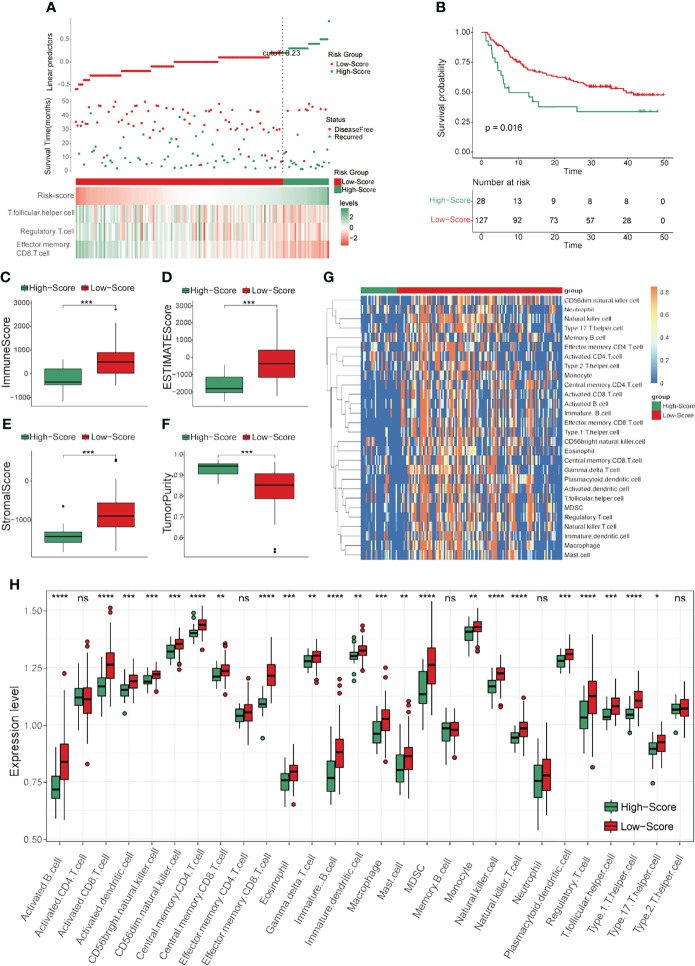
External validation of the TCRS efficiency in validation cohort 1. **(A)** Survival status and abundances effector memory of CD8 T cells, regulatory T cells and follicular helper T cells for the high- and low-score groups. **(B)** Kaplan-Meier curve for the two groups in the validation cohort 1. **(C–F)** The expression levels of the immune score **(C)**, ESTIMATE score **(D)**, stromal score **(E)** and tumor purity **(F)** for the two groups. **(G, H)** The expression levels of 28 immune cells of the two groups visualized by heatmap **(G)** or boxplot **(H)**. *p < 0.05, **p < 0.01, ***p < 0.001, ****p < 0.0001, ns: not significant.

To further validate the efficiency of the TCRS, two validation cohorts (validation cohort 1 and validation cohort 2) were analyzed in this study. The abundances of 28 immune cells were calculated by ssGSEA, and the TCRS was calculated using the same formula as in the training cohort. Next, HCC patients were also divided into high- and low-score groups according to the optimal cutoff values of the TCRS in the two validation cohorts. The group information, DFS status and abundances of effector memory CD8 T cells, regulatory T cells and follicular helper T cells between the high- and low-score groups were also visualized by ggrisk plots (**Figure 3A** and **Supplementary Figure 3A**). The results of survival analyses also showed that patients in the low-score group had a significantly longer DFS time than those in the high-score group in the two validation cohorts (**Figure 3B** and **Supplementary Figure 3B**), the corresponding AUCs for 1-, 3-, and 5-years DFS time prediction of the TCRS in these two validation cohorts were shown in **Supplementary Figure 1B**, **C**. In addition, the DFS time of disease-free patients were also significantly longer than that of recurrent patients in the two validation cohorts (**Supplementary Figure 2B, C**). Next, the results showed that the immune score, ESTIMATE score and stromal score were significantly higher in the low-score group (**Figure 3C–E** and **Supplementary Figure 3C–E**), while tumor purity was significantly higher in the high-score group (**Figure 3F** and **Supplementary Figure 3F**). Moreover, the majority of the 28 immune cells were also significantly higher in the low-score group (**Figure 3G, H** and **Supplementary Figure 3G, H**). These results suggested that the immune subtype of HCC could be well identified by the TCRS for prediction of DFS time.

### Differential Analyses Between the Two Groups Separated by TCRS

**Figure 4:**
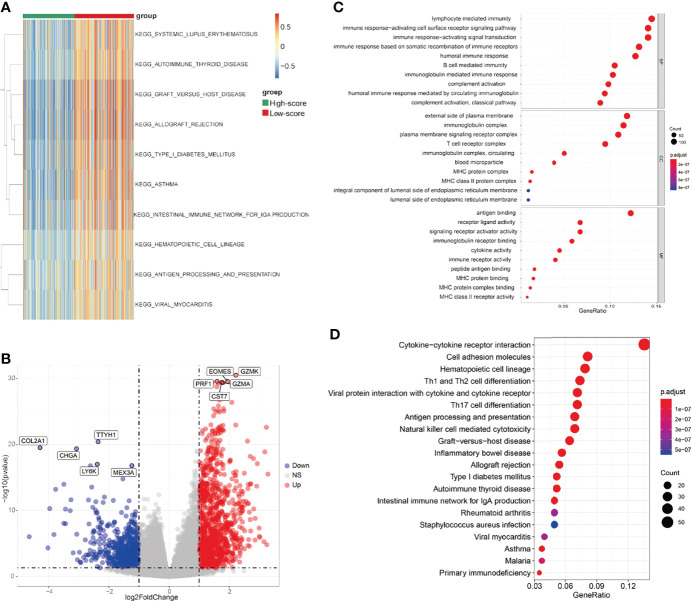
Differential analyses between the two groups separated by the TCRS in the training cohort. **(A)** The heatmap of the top ten significant terms from GSVA. **(B)** Volcano plot of the DEGs between the low- and high-score groups. DEGs were identified by comparing the count data of low- and high-score groups. P < 0.05 and |log2FoldChange| > 1 were identified as significant DEGs. Red dots represent significantly upregulated genes in the low-score group, blue dots represent significantly downregulated genes in the low-score group, while gray dots represent genes with no statistical difference. **(C)** Dot plot of GO results. BP: biological process; CC: cellular components; MF: molecular function. **(D)** Dot plot of KEGG results.

In order to clarify the functional differences between the high- and low-score groups, differential analyses were performed in the training cohort. First, GSVA was conducted to elucidate the general differences. As revealed by **Figure 4A**, the top ten most significantly different terms included many immune-related terms such as autoimmune diseases, intestinal immune network for IgA production and antigen processing and presentation. Next, DEGs between the two groups were identified and visualized by volcano plot (**Figure 4B**), and the significant DEGs were selected for further GO and KEGG analyses. The most enriched results in GO analysis were immune-related terms such as lymphocyte mediated immunity, immune response-activation signal transduction and humoral immune response in biological process (BP); immunoglobulin complex, T cell receptor complex and MHC protein complex in cellular components (CC); and antigen binding, receptor ligand activity and cytokine activity in molecular function (MF) (**Figure 4C**). In addition, the results of KEGG analysis showed that the most significantly different terms were also immune-related, such as cytokine-cytokine receptor interaction, Th1 and Th2 cell differentiation and antigen processing and presentation (**Figure 4D**). These results preliminarily identified the functional differences between the two risk score groups of HCC patients divided by the TCRS, and these differential terms may be potential targets for intervention to prolong DFS time.

### Identification of the Specific Targets for Recurrence Prevention by scRNA Sequencing Data of HCC

**Figure 5:**
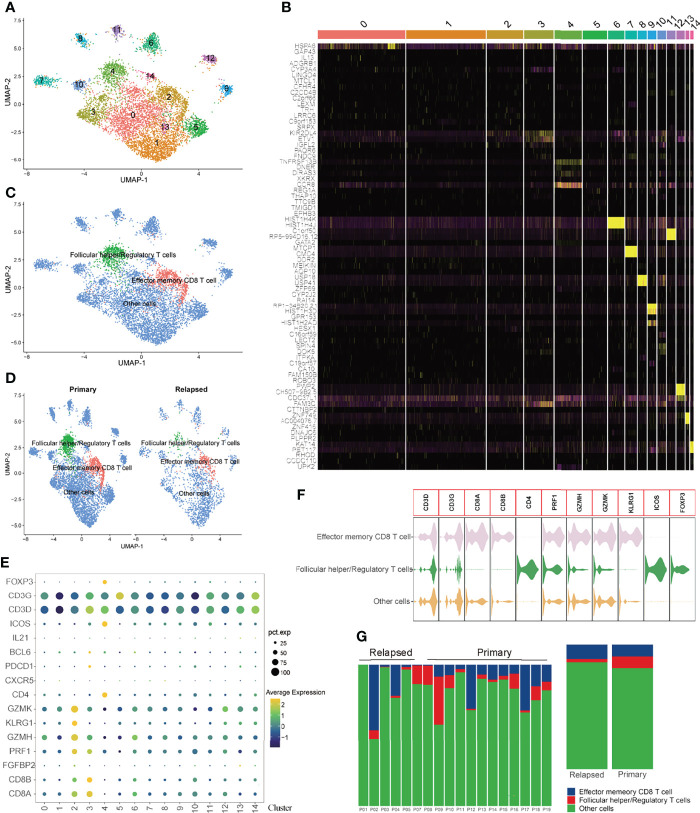
Visualization plots of T cell scRNA-seq data. **(A)** UMAP plot of 5415 cells from primary HCC and 1879 cells from relapsed HCC. **(B)** Heatmap of the top five significant DEGs in each cell cluster. **(C, D)** Annotations for three cell types. **(E)** Expression profiles of the marker genes in each cell cluster. **(F)** Violin plot of marker genes in each cell type. **(G)** Proportions of different T cell subtypes in each sample (left panel) or different tumor types (right panel).

To systematically assess the synergistic effect of the three prognostic immune cells in HCC recurrence, scRNA sequencing data were analyzed. Since the immune cells identified for the construction of the TCRS were both T cells, a total of 5415 T cells from primary HCC and 1879 T cells from relapsed HCC were analyzed in this study. The expression profiles of primary and relapsed tumor T cells, as well as the correlations between nFeature-RNA and nCount-RNA, are visualized in **Supplementary Figure 4A–B**. Next, fifteen T cell clusters were identified by UMAP analysis, and the top five significant DEGs in each cluster (**Supplementary Table 3**) were visualized by heatmap (**Supplementary Figure 4C** and **Figures 5A, B**). As revealed by **Supplementary Figures 4D–E**, there were no significant batch effects caused by the cell cycle or mitochondrial genes. Next, these T cell clusters were annotated with three cell types (effector memory CD8 T cells, follicular helper/regulatory T cells and other cells) according to the specific markers (**Figure 5C–E**), and the specific genes for each cell type were visualized by violin plot (**Figure 5F**). The proportions of different T cell subtypes in each sample or different tumor types are shown in **Figure 5G**.

**Figure 6:**
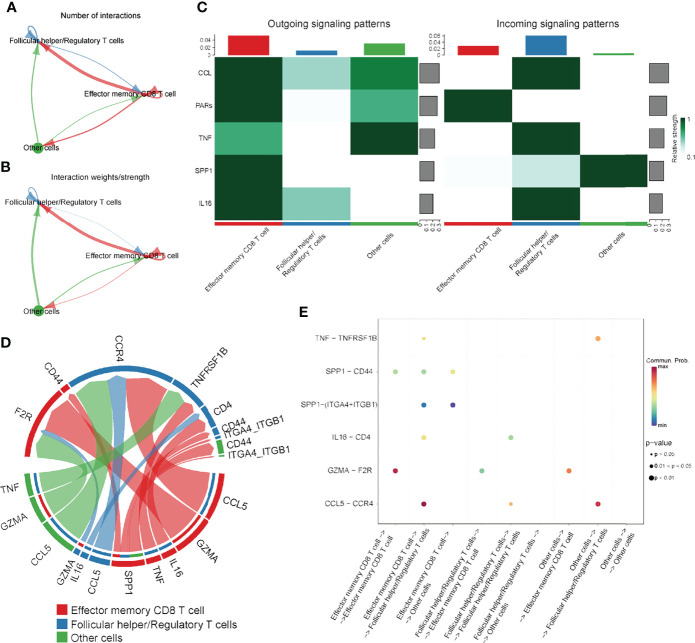
The results of cell-cell communication analysis. **(A)** Number of interactions among immune cells. **(B)** Strength of the interactions among the immune cells. **(C)** Heatmap visualizing the possible incoming or outgoing signaling pathways among the immune cells. **(D, E)** Chord diagram or dot plot visualizing the possible incoming or outgoing signaling pairs.

To further elucidate the integrated role of these immune cells, cell-cell communication analysis was performed. Interactions among these cell types are visualized in **Figure 6A–B**. Next, the potential outgoing and incoming signals, as well as the specific molecule pairs among these three cell types, were further investigated. As revealed by **Figure 6C**, effector memory CD8 T cells were the major signal provider, and follicular helper/regulatory T cells were the major signal receptor, while the potential signaling pathways among these cell types included CCL, PARs, TNF, SPP1 and IL16. Subsequently, the specific signal pairs among these cell types were investigated. The results showed that the strongest communication among these three cell types was from effector memory CD8 T cells to themselves through the GZMA-F2R (SPP1) signaling pathway, as well as effector memory CD8 T cells or other cells to follicular helper/regulatory T cells by the CCL5-CCR4 (CCL) signaling pathway (**Figures 6D, E**). These results preliminarily elucidated the potential interactions among these cell types, which was useful to for helping us further investigate the integrated role of effector memory CD8 T cells, regulatory T cells and follicular helper T cells in the DFS time prediction and recurrence prevention of HCC.

### Identification of the Specific Targets for Recurrence Prevention

**Figure 7:**
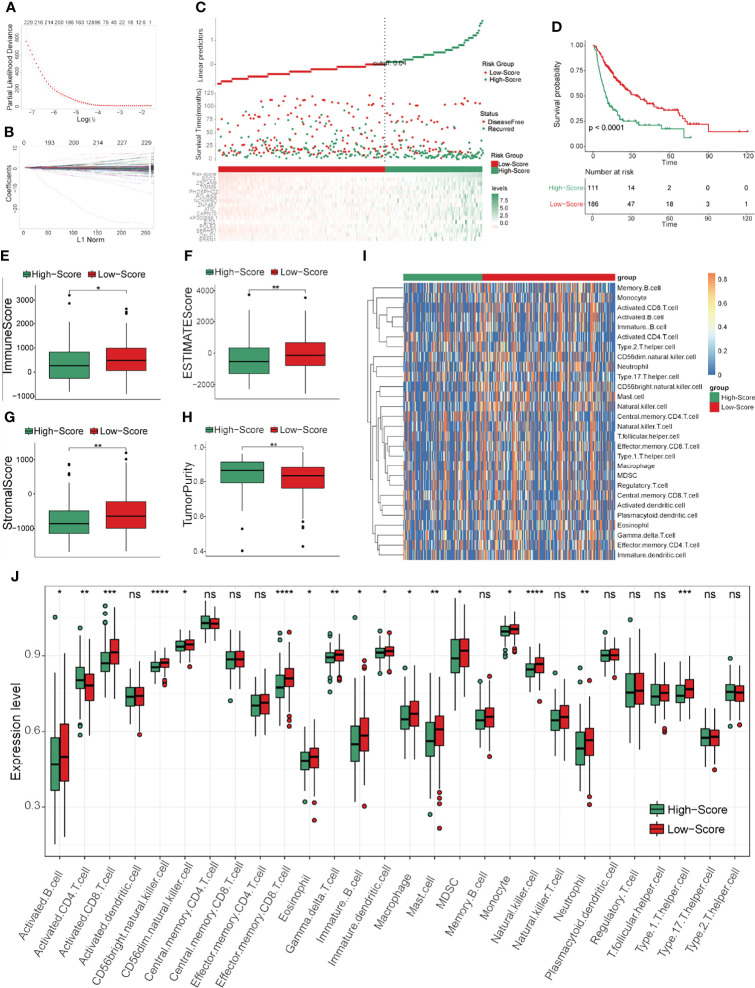
Construction and evaluation of GRS in the training cohort. **(A, B)** Cvfit and fit plots of LASSO screen. **(C)** Survival status and the expression levels of fifteen genes in the high- and low-score groups separated by GRS. **(D)** Kaplan-Meier curve for the two groups in the training cohort. **(E–H)** The expression levels of the immune score **(E)**, ESTIMATE score **(F)**, stromal score **(G)** and tumor purity **(H)** in the two groups. **(I, J)** The expression levels of 28 immune cells in the two groups visualized by heatmap **(I)** or boxplot **(J)**. *p < 0.05, **p < 0.01, ***p < 0.001, ****p < 0.0001, ns: not significant.

**Figure 8:**
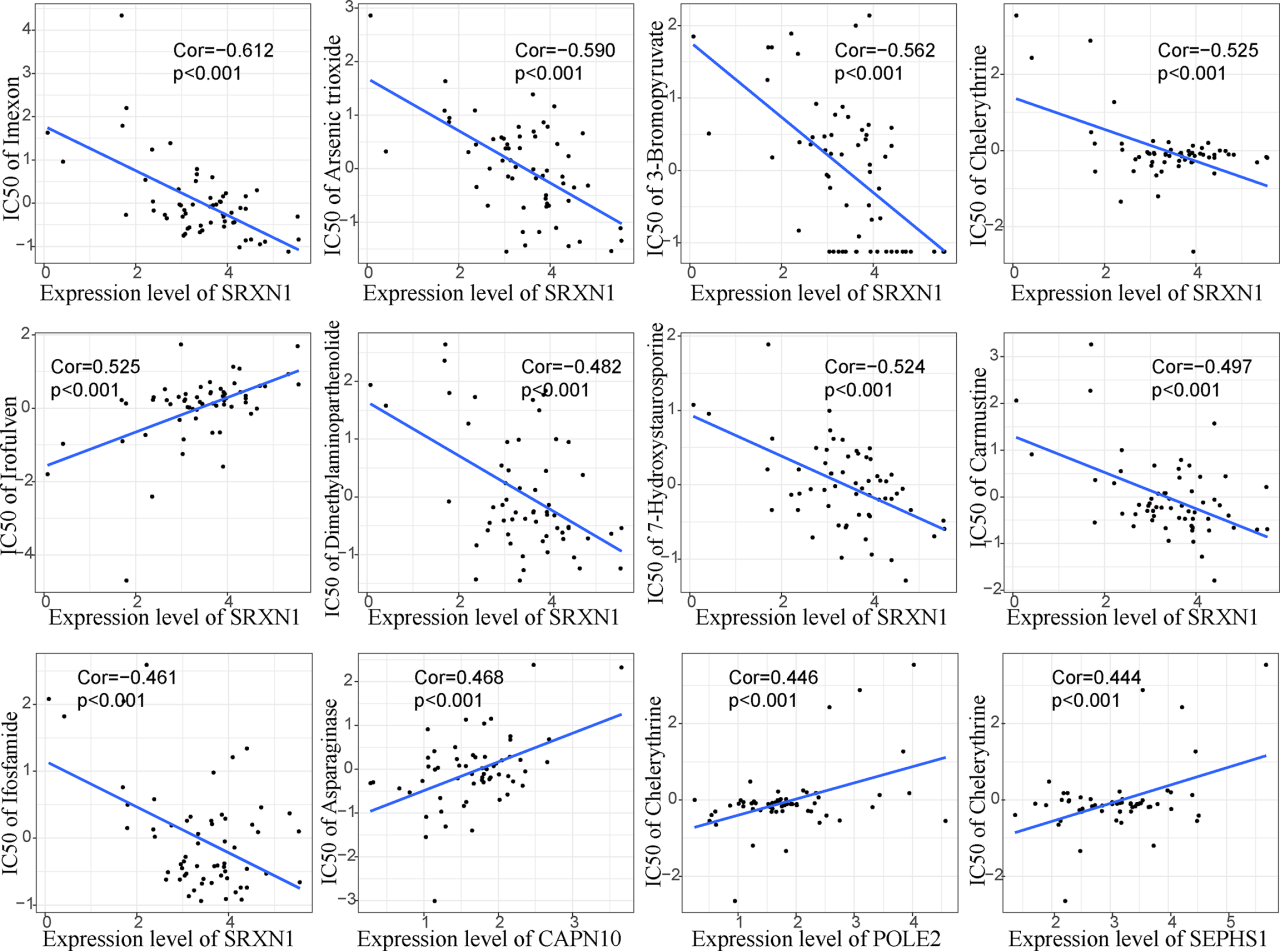
Pearson correlation analysis between the fifteen prognostic genes and their potential targeted drugs. Twelve representative scatter plots of the relationship between drug sensitivity (IC50) and the expression levels of the fifteen hub genes. Positive correlation means that with the increase of gene expression level, the IC50 value of drugs also increases, and vice versa.

To further screen the hub genes in prognostic immune cells that might associated with HCC recurrence, DEGs between primary and relapsed HCC were identified using scRNA data. As a result, a total of 298 and 407 DEGs were identified in the effector memory CD8 T cells and follicular helper/regulatory T cells, respectively, and 645 genes remained for further analyses by generating union sets. After screening by Cox and subsequent LASSO analyses (**Supplementary Table 4** and **Figures 7A, B**), fifteen prognostic genes (AP000866.1, ATIC, CAPN10, EDC3, EID3, NCKIPSD, OXLD1, PHOSPHO2, POLE2, POLR3G, SEPHS1, SRXN1, TIMM9, ZNF487 and ZSCAN9) were identified. We note that most of these genes exhibited significantly high expression in the high-TCRS group, except for AP000866.1, EID3, POLR3G and ZNF487 (**Supplementary Figures 5A–O**). Next, a GRS was constructed from the expression levels of these fifteen prognostic genes and their corresponding coefficients (**Supplementary Table 5**). The group information, DFS status and expression levels of these fifteen hub genes for high- and low-score groups, defined by applying the optimal cutoff value of GRS, are shown in **Figure 7C**. The results of survival analyses showed that patients in the low-score group had a significantly longer DFS time than those in the high-score group (**Figures 7C, D**). The AUCs for 1-, 3-, and 5-years DFS time prediction of the GRS were 0.74, 0.68, and 0.74, respectively (**Supplementary Figure 1D**). In addition, the results of immune analyses showed that the low-GRS group could also be well identified as an immune subtype with a longer DFS time and inflammatory immune characteristics, consistent with the results of the TCRS (**Figures 7E–J**). These results indicated that these fifteen hub genes may be related to the process of immune cells affecting DFS time, which may also be potential targets for preventing recurrence of HCC. Finally, the potential drugs for preventing recurrence were preliminarily screened by CellMiner (**Supplementary Table 6**). After comparing the expression levels of the hub genes and the IC50 values of drugs that have been approved by the Food and Drug Administration (FDA) or are in clinical trials, the twelve most significant correlation pairs are visualized in **Figure 8**, such as the irofulven-SRXN1 and chelerythrine-POLE2 pairs. These results implied that these drugs may be effective in the prevention of recurrence in HCC patients by targeting the fifteen hub genes.

## Discussion

At present, radical surgery is the best treatment for HCC patients among surgical options, with the five-year survival rate reaching more than 70% (6). However, approximately 70% of HCC patients who undergo radical surgery develop tumor recurrence within five years postsurgery (6, 40). For this reason, it is particularly important to construct efficient models to predict DFS time and find specific targets for the prevention of recurrence to improve the treatment.

In this study, an efficient immune-related risk score (TCRS) was constructed based on three immune cell types (effector memory CD8 T cells, regulatory T cells and follicular helper T cells) to identify the immune subtype of HCC patients with longer DFS times and inflammatory immune characteristics. It is noteworthy that although most immune cell types are highly expressed in the immune subtype of HCC, some of them are adverse prognostic factors such as regulatory T cells and follicular helper T cells with risk coefficients greater than zero. In addition, the result of GSVA showed that immune related pathways were significantly enriched in the low-TCRS group (**Figure 4A**), including autoimmunity (such as systemic lupus erythematosus) and adaptive immune response processes (such as antigen processing and presentation). It is controversial that many studies have shown that autoimmunity inhibits the function of the immune system, while antigen presentation helps to improve the immune response (41–44). However, these results suggest that the inflammatory immune microenvironment in the low-score group as a whole plays a protective role in prolonging the DFS time of HCC.

Among these three immune cell types, effector memory CD8 T cells were the only protective factor with a risk coefficient less than zero. Effector memory CD8 T cells have been reported to play a role in IL-15 signaling-related antitumor activity of PD-1 inhibitors, as well as prognosis prediction of gastric cancer, lung cancer and ovarian cancer (45–48). However, its role in prognosis prediction and cancer recurrence remains largely unknown.

Regulatory T cells have been widely recognized as immune-suppressive cells in a variety of cancers, and targeting regulatory T cells has become a promising approach in cancer immunotherapy (49–54). However, the role of regulatory T cells in HCC recurrence after surgery is still controversial. For example, a recent study showed that the expression of regulatory T cells decreased in early-relapse HCC (25), but it has also been reported that CXCL10/CXCR3 signaling or deficits in CD4 cytotoxic T cells could induce regulatory T cell mobilization to promote liver tumor recurrence (55, 56). In addition, a combination of depletion of regulatory T cells and concomitant stimulation of effector T cells has been recommended as an immunotherapy method for reducing the chance of HCC recurrence after surgery (57).

Studies have shown that follicular helper T cells play a role in liver cirrhosis and autoimmune liver diseases (58–60). In addition, follicular helper T cells combined with regulatory T cells have been reported to participate in the response to immunotherapy in TP53-mutated HCC patients (61). However, whether follicular helper T cells play a role in HCC recurrence remains unclear.

In this study, the integrated role of these three immune cell types in the prediction DFS time was reported for the first time. In addition, functional differences between the high- and low-score groups were clarified, and cell-cell communication among these immune cells was preliminarily elucidated by scRNA sequencing data. These results will further guide us in finding methods to prolong DFS time by targeting these immune cells.

With the development of high-throughput sequencing technology, combined analysis of multi-omics data has become an effective method to comprehensively clarify disease heterogeneity, predict disease prognosis and find new therapeutic targets (62–65). To further address the specific targets for HCC recurrence prevention, bulk- and scRNA sequencing data were integrated analyzed. At first, DEGs between primary and relapsed HCC were identified in effector memory CD8 T cells or follicular helper/regulatory T cells by scRNA data. After survival and immune analysis, fifteen prognostic DEGs were screened out and a GRS was constructed. These results suggested that these hub genes may be specific targets that interfere with the functions of the three prognostic immune cell types.

CellMiner is a web-based tool for screening therapeutic drugs targeting specific genes (39). To search for potential targets for the prevention of recurrence, correlations between the fifteen hub genes and the IC50 of drugs were analyzed in this study (**Supplementary Table 6**). The results suggest that postoperative treatment with these drugs, such as imexon, irofulven and nelarabine, may delay the recurrence of HCC.

There are some limitations in this study. First, the time from operation to the last follow-up was defined as DFS time if the patient did not relapse during postoperative follow-up. A patient that is disease-free might have a shorter DFS time due to a lack of a determined maximum time without disease (such as a set time of 2 years). This may lead to deviations in the accuracy of the models. Second, the prognostic value of the TCRS should be validated in more data from additional HCC patients in real-world muticenter studies. Third, how to comprehensively affect the function of these three prognostic immune cells needs to be further studied. Fourth, whether the drugs targeting these fifteen hub genes can delay the recurrence of HCC remains to be confirmed by further experimental and clinical studies.

## Conclusions

In this study, a novel efficient TCRS was constructed to identify the immune subtype of HCC patients with longer DFS times and inflammatory immune characteristics. Functional differences between the high- and low-score groups separated by the TCRS were clarified, and cell-cell communication among these immune cells was preliminarily studied. Moreover, fifteen hub genes that might associated with the roles of the TCRS in HCC were identified by scRNA sequencing data, indicating that they may be potential therapeutic targets for the prevention of HCC recurrence.

## Data Availability Statement

The datasets presented in this study can be found in online repositories. The names of the repository/repositories and accession number(s) can be found in the article/**Supplementary Material**.

## Author Contributions

JF and XL conceived the study and analyzed the data. All authors drafted the article and approved the final manuscript.

## Funding

This work was supported by the Scientific Research Fund Project of Hunan Provincial Health Commission (20200284).

## Conflict of Interest

The authors declare that the research was conducted in the absence of any commercial or financial relationships that could be construed as a potential conflict of interest.

## Publisher’s Note

All claims expressed in this article are solely those of the authors and do not necessarily represent those of their affiliated organizations, or those of the publisher, the editors and the reviewers. Any product that may be evaluated in this article, or claim that may be made by its manufacturer, is not guaranteed or endorsed by the publisher.

## Supplementary Material

Supplementary Figure 1 Time-dependent ROC curves in different cohorts. **(A)** ROC curves of the TCRS in the training cohort. **(B)** ROC curves of the TCRS in the validation cohort 1. **(C)** ROC curves of the TCRS in the validation cohort 2 (GSE14520). **(D)** ROC curves of the GRS in the training cohort.

Supplementary Figure 2 Comparison of DFS time between patients with disease-free and recurrent HCC. **(A)** Comparison results in the training cohort. **(B)** Comparison results in the validation cohort 1. **(C)** Comparison results in the validation cohort 2 (GSE14520).

Supplementary Figure 3 External validation of the TCRS efficiency in validation cohort 2 (GSE14520). **(A)** Survival status and abundances of effector memory CD8 T cells, regulatory T cells and follicular helper T cells between the high- and low-score groups. **(B)** Kaplan-Meier curve for the two groups in the validation cohort 2. **(C–F)** The expression levels of the immune score **(C)**, ESTIMATE score **(D)**, stromal score **(E)** and tumor purity **(F)** between the two groups. **(G, H)** The expression levels of 28 immune cells of the two groups visualized by heatmap **(G)** or boxplot **(H)**. *p < 0.05, **p < 0.01, ***p < 0.001, ****p < 0.0001, ns: not significant.

Supplementary Figure 4 Quality control of the HCC scRNA data. **(A)** Expression profiles of T cells from primary and relapsed HCC samples. **(B)** Correlations between nFeature-RNA and nCount-RNA (left panel) or nFeature-RNA and percent.mt (right panel) in T cells. **(C)** UMAP plot separated by tissue types. **(D)** UMAP plot separated by cell cycle. **(E)** UMAP plot showing the expression levels of mitochondrial genes.

Supplementary Figure 5 The expression levels of the fifteen prognostic genes constructed for the TCRS between the high- and low-score groups in the training cohort. *p < 0.05, **p < 0.01, ***p < 0.001, ns: not significant.
