# Supplementary figures and images for: Identification of the Immune Subtype of Hepatocellular Carcinoma for the Prediction of Disease-Free Survival Time and Prevention of Recurrence by Integrated Analysis of Bulk- and Single-Cell RNA Sequencing Data
Source: Front Immunol. 2022 Jun 6;13:868325. doi: 10.3389/fimmu.2022.868325 (PMC9207181; doi:10.3389/fimmu.2022.868325)

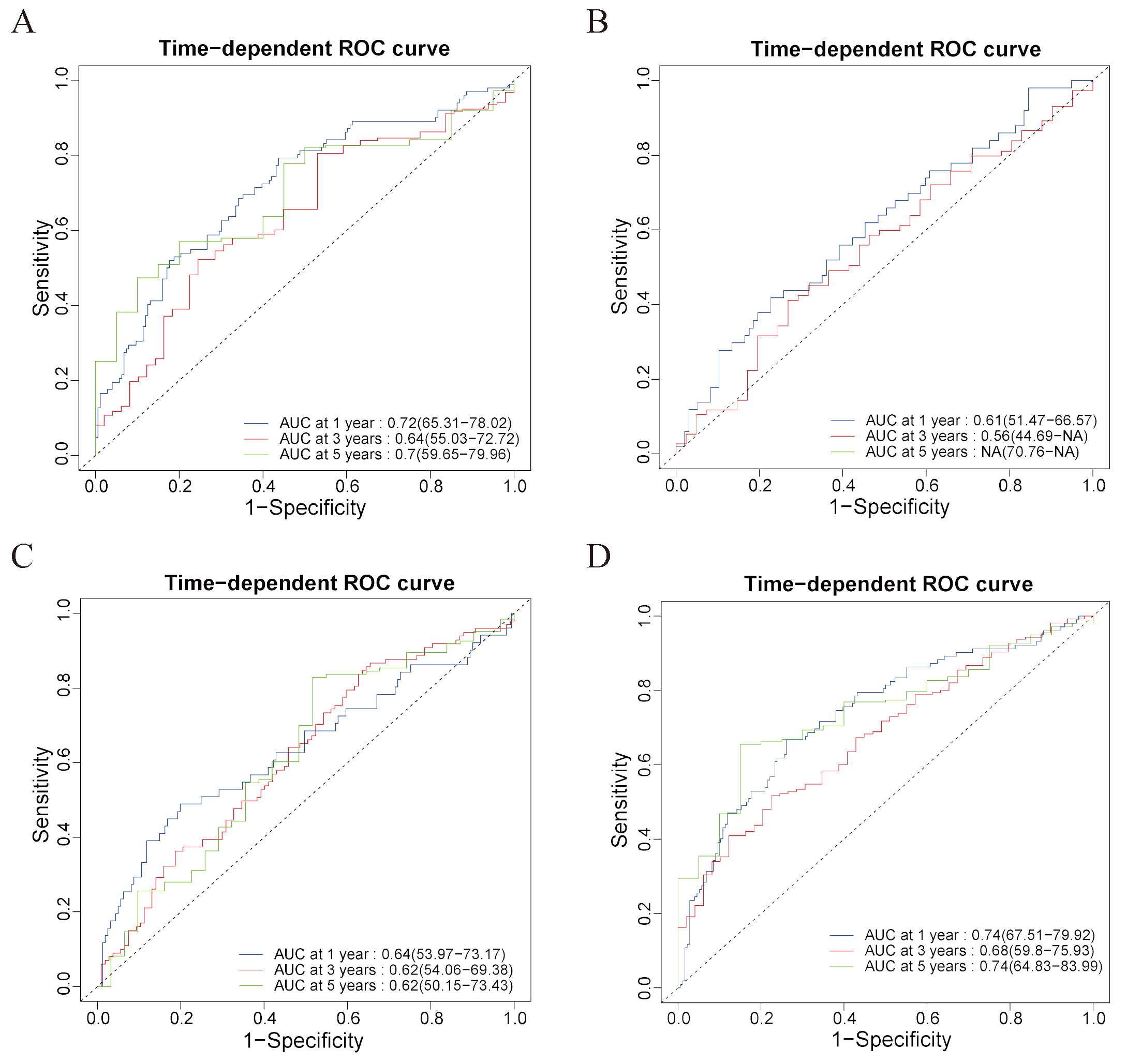

Supplement: Supplementary Figure 1 — Time-dependent ROC curves in different cohorts. (A) ROC curves of the TCRS in the training cohort. (B) ROC curves of the TCRS in the validation cohort 1. (C) ROC curves of the TCRS in the validation cohort 2 (GSE14520). (D) ROC curves of the GRS in the training cohort. [file Image_1.tif]

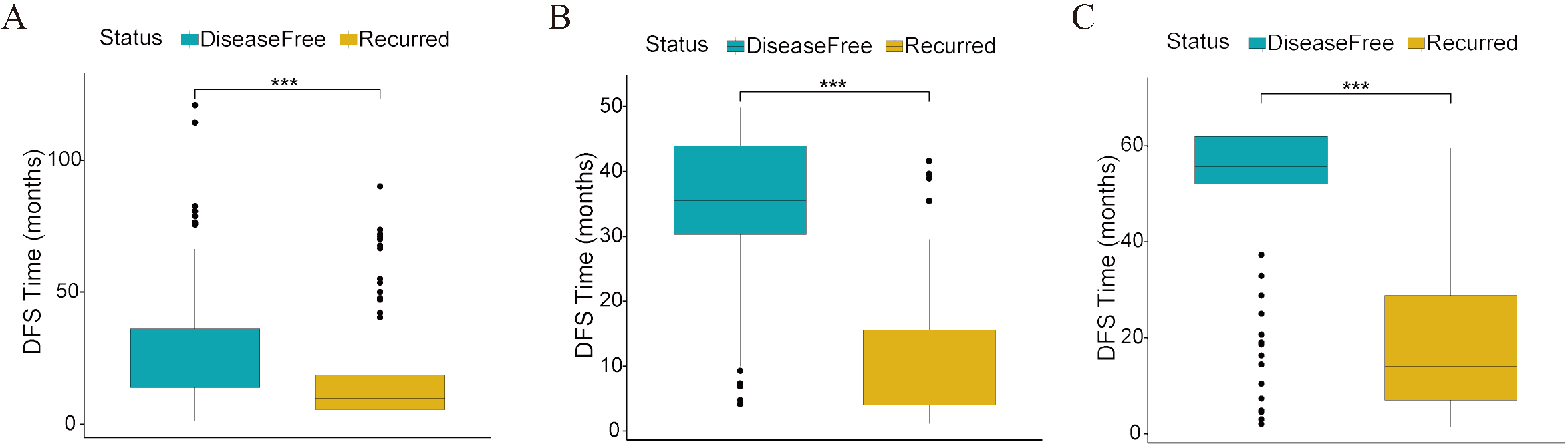

Supplement: Supplementary Figure 2 — Comparison of DFS time between patients with disease-free and recurrent HCC. (A) Comparison results in the training cohort. (B) Comparison results in the validation cohort 1. (C) Comparison results in the validation cohort 2 (GSE14520). [file Image_2.tif]

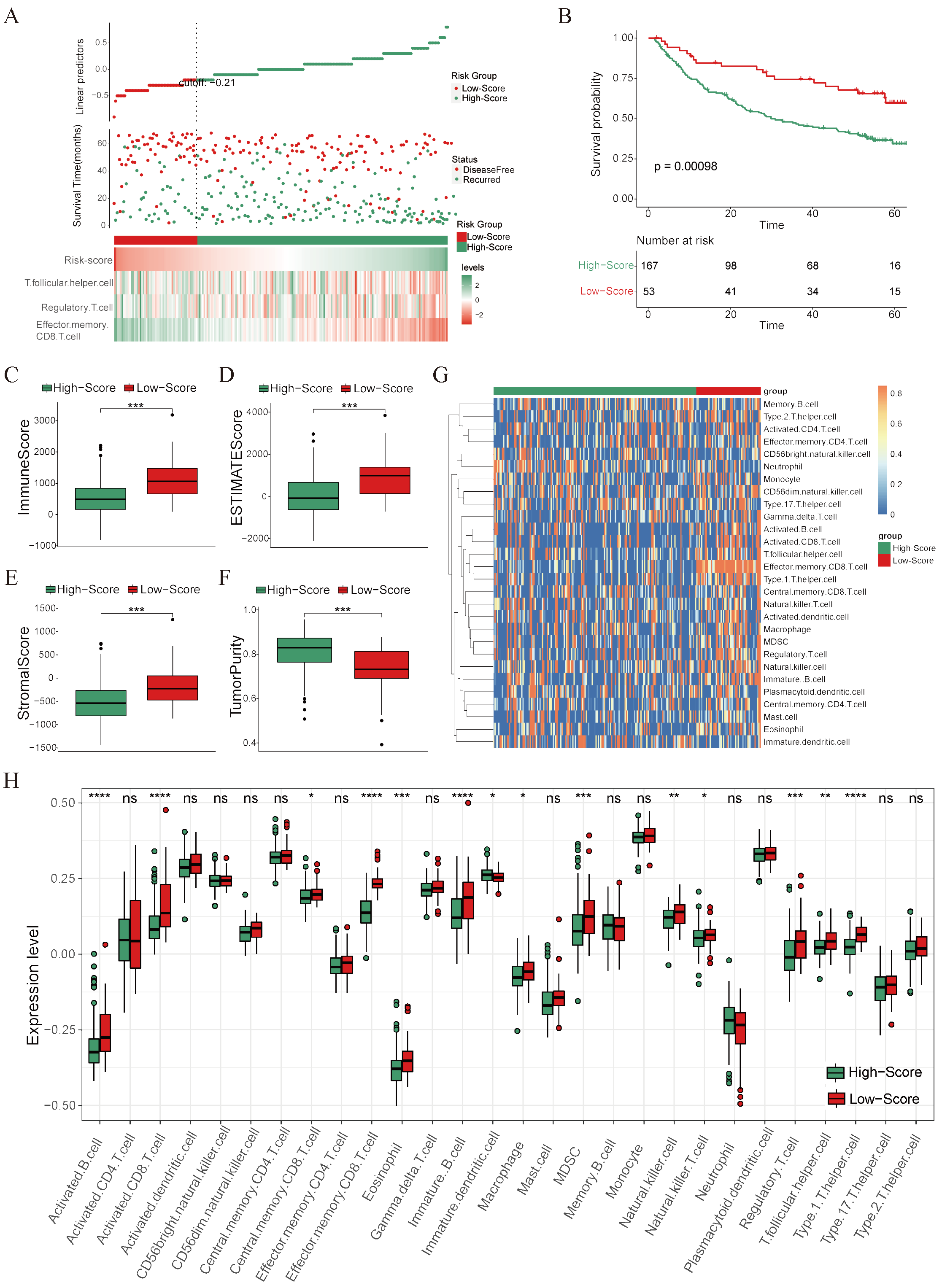

Supplement: Supplementary Figure 3 — External validation of the TCRS efficiency in validation cohort 2 (GSE14520). (A) Survival status and abundances of effector memory CD8 T cells, regulatory T cells and follicular helper T cells between the high- and low-score groups. (B) Kaplan-Meier curve for the two groups in the validation cohort 2. (C–F) The expression levels of the immune score (C), ESTIMATE score (D), stromal score (E) and tumor purity (F) between the two groups. (G, H) The expression levels of 28 immune cells of the two groups visualized by heatmap (G) or boxplot (H). *p < 0.05, **p < 0.01, ***p < 0.001, ****p < 0.0001, ns: not significant. [file Image_3.tif]

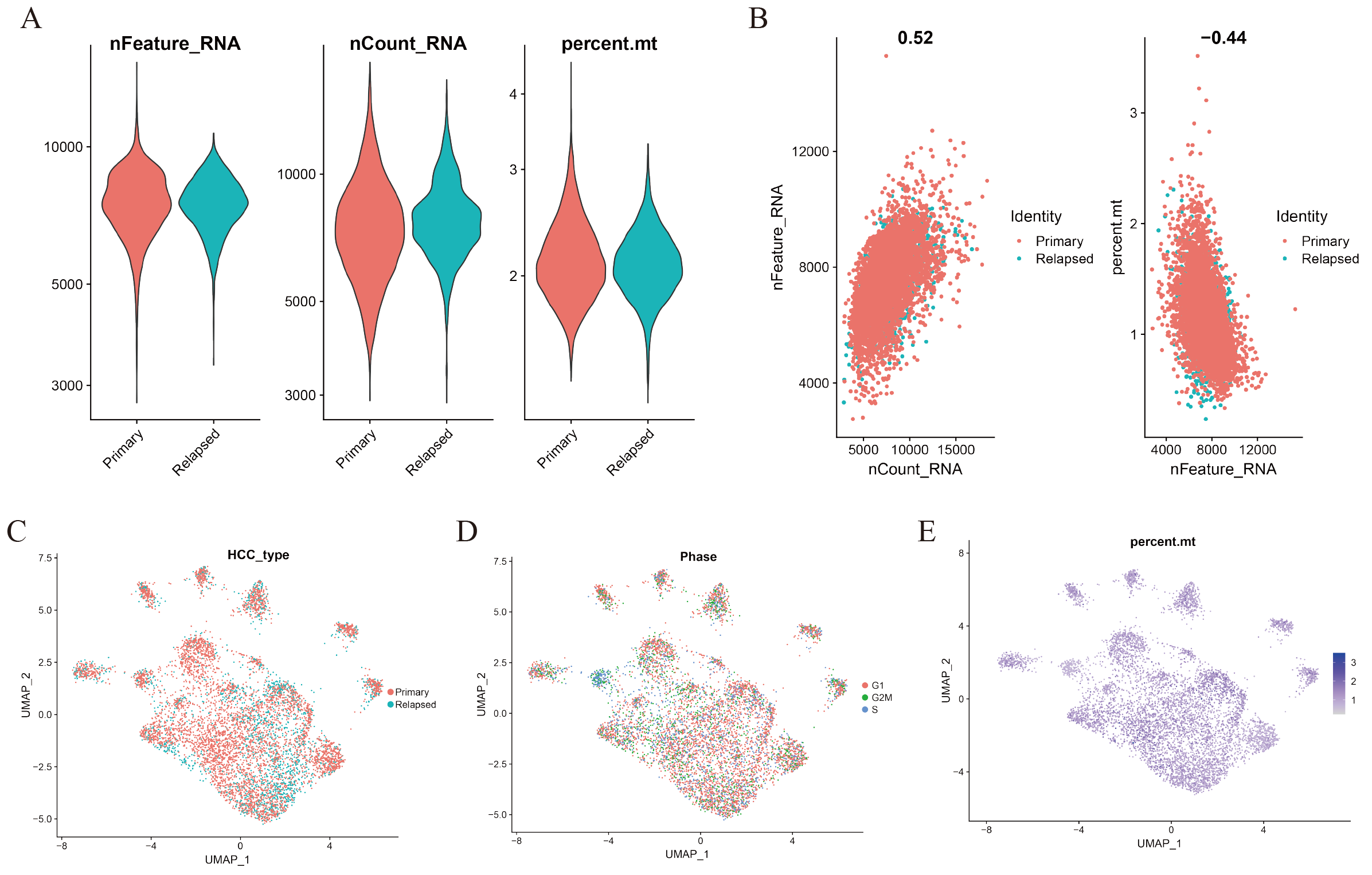

Supplement: Supplementary Figure 4 — Quality control of the HCC scRNA data. (A) Expression profiles of T cells from primary and relapsed HCC samples. (B) Correlations between nFeature-RNA and nCount-RNA (left panel) or nFeature-RNA and percent.mt (right panel) in T cells. (C) UMAP plot separated by tissue types. (D) UMAP plot separated by cell cycle. (E) UMAP plot showing the expression levels of mitochondrial genes. [file Image_4.tif]

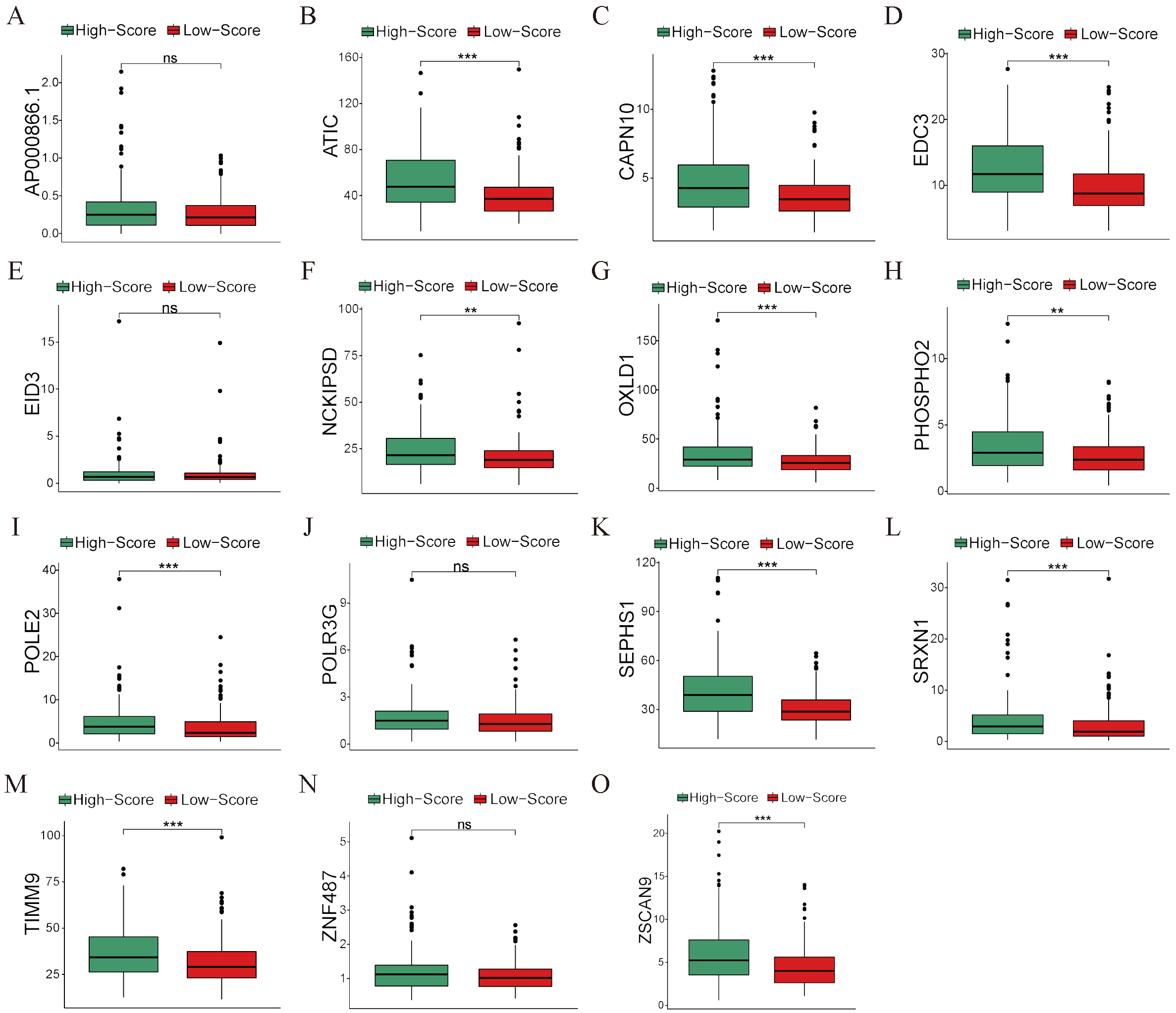

Supplement: Supplementary Figure 5 — The expression levels of the fifteen prognostic genes constructed for the TCRS between the high- and low-score groups in the training cohort. *p < 0.05, **p < 0.01, ***p < 0.001, ns: not significant. [file Image_5.tif]
