# Supplementary material for: Identification of the Immune Subtype of Hepatocellular Carcinoma for the Prediction of Disease-Free Survival Time and Prevention of Recurrence by Integrated Analysis of Bulk- and Single-Cell RNA Sequencing Data
Source: Front Immunol. 2022 Jun 6;13:868325. doi: 10.3389/fimmu.2022.868325 (PMC9207181; doi:10.3389/fimmu.2022.868325)
Supplement: Supplementary file 7 [file Table_2.docx]

Table S2. Specific markers for cell annotation.

| Cell Type | Cell Marker |
| --- | --- |
| Follicular helper T cells | CD4, CXCR5, PDCD1, BCL6, IL21, ICOS |
| Regulatory T cells | CD4, CD25, FOXP3 |
| Effector memory CD8 T cells | FGFBP2, PRF1, GZMH, KLRG1, GZMK |
| CD8 T cells | CD8A, CD8B |
| Other cells | CD3D, CD3G |
