# Supplementary material for: Identification of the Immune Subtype of Hepatocellular Carcinoma for the Prediction of Disease-Free Survival Time and Prevention of Recurrence by Integrated Analysis of Bulk- and Single-Cell RNA Sequencing Data
Source: Front Immunol. 2022 Jun 6;13:868325. doi: 10.3389/fimmu.2022.868325 (PMC9207181; doi:10.3389/fimmu.2022.868325)
Supplement: Supplementary file 8 [file Table_3.docx]

Table S3. Top 5 significant DEGs in each cell cluster.

| Cluster | Gene | Adjust p value |
| --- | --- | --- |
| 0 | HSPA6 | 1.32E-100 |
| 0 | GAP43 | 4.68E-95 |
| 0 | IL13 | 2.58E-37 |
| 0 | ADGRB1 | 0.181484612 |
| 0 | CYP3A4 | 1 |
| 1 | LINGO4 | 3.61E-63 |
| 1 | MTCL1 | 2.47E-55 |
| 1 | CFHR4 | 1.01E-36 |
| 1 | C2CD4B | 4.31E-12 |
| 1 | C2orf66 | 0.004612594 |
| 2 | LEXM | 5.42E-56 |
| 2 | TRH | 2.11E-20 |
| 2 | LRRC6 | 2.02E-13 |
| 2 | C9orf153 | 9.70E-13 |
| 2 | SRPX | 1 |
| 3 | KIR2DL4 | 1.86E-117 |
| 3 | ETV1 | 9.26E-35 |
| 3 | IGFL2 | 9.06E-23 |
| 3 | PAQR6 | 1.80E-07 |
| 3 | FNDC9 | 3.32E-07 |
| 4 | TNFRSF13B | 4.41E-199 |
| 4 | DNER | 3.09E-83 |
| 4 | DIRAS3 | 1.01E-31 |
| 4 | XKRX | 1.24E-30 |
| 4 | CCR8 | 0.334639212 |
| 5 | REG1A | 1.99E-119 |
| 5 | THAP10 | 1.04E-99 |
| 5 | TTC9B | 3.26E-95 |
| 5 | TMIGD1 | 2.93E-53 |
| 5 | EPHB3 | 7.64E-50 |
| 6 | HIST1H4K | 2.16E-197 |
| 6 | HIST1H4J | 7.59E-197 |
| 6 | C1orf50 | 9.35E-74 |
| 6 | RP5-994D16.12 | 1.40E-53 |
| 6 | GATA2 | 6.92E-13 |
| 7 | MTCP1 | 4.97E-144 |
| 7 | CMC4 | 4.38E-139 |
| 7 | DDR2 | 3.13E-15 |
| 7 | MEIKIN | 2.07E-08 |
| 7 | AQP10 | 4.62E-06 |
| 8 | USP18 | 5.26E-108 |
| 8 | USP41 | 1.96E-99 |
| 8 | ZFP69 | 9.16E-13 |
| 8 | CYP2J2 | 0.036282928 |
| 8 | RAI14 | 0.041518233 |
| 9 | RP1-34B20.21 | 1.95E-108 |
| 9 | HIST1H3D | 3.57E-61 |
| 9 | GPR153 | 1.49E-19 |
| 9 | HIST1H2AD | 7.00E-06 |
| 9 | HESX1 | 1 |
| 10 | C16orf59 | 3.17E-53 |
| 10 | LECT2 | 5.92E-44 |
| 10 | SPIN4 | 3.54E-41 |
| 10 | DOK5 | 1.89E-15 |
| 10 | ITPKA | 2.68E-12 |
| 11 | RP5-994D16.12 | 1.63E-103 |
| 11 | C19orf57 | 6.61E-17 |
| 11 | CA10 | 3.07E-14 |
| 11 | FAM150B | 2.98E-09 |
| 11 | ROBO3 | 1 |
| 12 | PWP2 | 6.65E-101 |
| 12 | CH507-9B2.5 | 1.90E-98 |
| 12 | CDC37L1 | 9.93E-48 |
| 12 | FAM3C | 1.64E-09 |
| 12 | CTTNBP2 | 0.045283776 |
| 13 | ZNF749 | 5.77E-44 |
| 13 | AC004076.7 | 1.77E-42 |
| 13 | ZNF416 | 1.85E-13 |
| 13 | DNAJC6 | 0.000389306 |
| 13 | PLPPR2 | 0.021553861 |
| 14 | KAT14 | 6.45E-36 |
| 14 | PET117 | 7.07E-35 |
| 14 | RHOD | 1 |
| 14 | CCDC110 | 1 |
| 14 | UPK2 | 1 |
