# Supplementary material for: Identification of the Immune Subtype of Hepatocellular Carcinoma for the Prediction of Disease-Free Survival Time and Prevention of Recurrence by Integrated Analysis of Bulk- and Single-Cell RNA Sequencing Data
Source: Front Immunol. 2022 Jun 6;13:868325. doi: 10.3389/fimmu.2022.868325 (PMC9207181; doi:10.3389/fimmu.2022.868325)
Supplement: Supplementary file 9 [file Table_4.docx]

Table S4. Univariate and multivariate Cox regression analyses of the 645 DEGs.

| Gene | Univariate analysis | | | Multivariate analysis | | |
| --- | --- | --- | --- | --- | --- | --- |
|  | HR | 95% CI | P value | HR | 95% CI | P value |
| AADAC | 1 | 1.00-1.00 | 0.119 | NA | NA | NA |
| ABCD3 | 1 | 0.99-1.01 | 0.822 | NA | NA | NA |
| ABCF3 | 1.02 | 1.01-1.04 | 0.008 | 0.96 | 0.94-0.98 | 0 |
| ABHD4 | 1.01 | 1-1.01 | 0.029 | 0.92 | 0.91-0.93 | 0 |
| ABHD6 | 1 | 0.98-1.01 | 0.389 | NA | NA | NA |
| AC006116.20 | 1.14 | 0.52-2.52 | 0.742 | NA | NA | NA |
| AC129492.1 | NA | NA-NA | NA | NA | NA | NA |
| ACCS | 1.03 | 0.98-1.08 | 0.195 | NA | NA | NA |
| ACVR1 | 1.01 | 1-1.02 | 0.038 | 0.94 | 0.93-0.96 | 0 |
| ACVR1B | 1.01 | 1-1.02 | 0.085 | NA | NA | NA |
| ADAT3 | 1.18 | 1.04-1.33 | 0.008 | 0.97 | 0.84-1.12 | 0.718 |
| ADHFE1 | 0.99 | 0.97-1 | 0.009 | 1 | 0.99-1.01 | 0.96 |
| ADO | 1.04 | 1.02-1.07 | 0.002 | 3.07 | 2.96-3.18 | 0 |
| ADORA2A | 1.25 | 1.06-1.47 | 0.008 | 0.65 | 0.55-0.77 | 0 |
| ADRA2B | 0.96 | 0.84-1.1 | 0.586 | NA | NA | NA |
| AHCYL2 | 1.03 | 0.99-1.08 | 0.133 | NA | NA | NA |
| AHSP | 0.93 | 0.56-1.54 | 0.769 | NA | NA | NA |
| ALG6 | 1.07 | 1.03-1.12 | 0.002 | 2.23 | 2.11-2.35 | 0 |
| ALKBH2 | 1.01 | 1-1.02 | 0.013 | 0.97 | 0.96-0.98 | 0 |
| ALKBH3 | 1.02 | 1-1.04 | 0.126 | NA | NA | NA |
| AMIGO3 | 38.72 | 1.02-1475.36 | 0.049 | 1.97E+16 | 3.13E+14-1.23E+18 | 0 |
| ANKAR | 1.43 | 1.07-1.9 | 0.015 | 2546.88 | 1716.41-3779.16 | 0 |
| ANKDD1B | 1.08 | 0.94-1.25 | 0.28 | NA | NA | NA |
| ANKMY2 | 1.03 | 1.01-1.05 | 0.009 | 1.43 | 1.4-1.47 | 0 |
| ANKRA2 | 1 | 0.98-1.03 | 0.782 | NA | NA | NA |
| ANKRD18A | 1.39 | 1.13-1.7 | 0.002 | 0.01 | 0-0.01 | 0 |
| ANO9 | 1.03 | 1-1.06 | 0.068 | NA | NA | NA |
| AP000866.1 | 2.4 | 1.57-3.66 | 0 | 14.97 | 9.02-24.83 | 0 |
| AP1S1 | 1 | 1-1.01 | 0.08 | NA | NA | NA |
| APBB1 | 1.03 | 1-1.05 | 0.07 | NA | NA | NA |
| APBB3 | 1.01 | 0.98-1.03 | 0.588 | NA | NA | NA |
| APLF | 1.17 | 0.98-1.39 | 0.077 | NA | NA | NA |
| APPL2 | 1.01 | 0.99-1.02 | 0.362 | NA | NA | NA |
| ARHGAP10 | 1.03 | 0.93-1.14 | 0.596 | NA | NA | NA |
| ARHGAP32 | 1.1 | 1.01-1.19 | 0.024 | 1.09 | 0.99-1.2 | 0.074 |
| ARHGAP33 | 1.08 | 1.04-1.12 | 0 | 0.23 | 0.22-0.24 | 0 |
| ARHGEF19 | 1.04 | 0.98-1.1 | 0.241 | NA | NA | NA |
| ARRDC1 | 1.02 | 1.01-1.04 | 0.006 | 1.13 | 1.11-1.15 | 0 |
| ASB1 | 1.05 | 1.01-1.09 | 0.011 | 0.75 | 0.71-0.8 | 0 |
| ASMTL | 1 | 0.99-1.01 | 0.42 | NA | NA | NA |
| ASTL | 2.74 | 0.79-9.48 | 0.111 | NA | NA | NA |
| ATF1 | 1.03 | 1.01-1.06 | 0.003 | 0.87 | 0.85-0.89 | 0 |
| ATIC | 1.01 | 1.01-1.02 | 0 | 1.14 | 1.13-1.15 | 0 |
| ATP10D | 1.05 | 0.99-1.12 | 0.099 | NA | NA | NA |
| ATPAF1 | 1.01 | 0.99-1.02 | 0.289 | NA | NA | NA |
| B3GNT8 | 1.02 | 0.99-1.05 | 0.168 | NA | NA | NA |
| B4GALT7 | 1.03 | 1.01-1.05 | 0.012 | 1.14 | 1.11-1.16 | 0 |
| BBS7 | 1.17 | 1.07-1.27 | 0 | 0.09 | 0.08-0.1 | 0 |
| BBS9 | 1.13 | 1.04-1.23 | 0.005 | 47.64 | 42.44-53.48 | 0 |
| BCKDHA | 0.98 | 0.94-1.03 | 0.441 | NA | NA | NA |
| BCORL1 | 1.07 | 1.03-1.1 | 0 | 0.56 | 0.53-0.59 | 0 |
| BFSP2 | 1.01 | 0.79-1.3 | 0.922 | NA | NA | NA |
| BMP4 | 1 | 0.99-1.01 | 0.967 | NA | NA | NA |
| BTBD6 | 1 | 0.99-1.01 | 0.853 | NA | NA | NA |
| BYSL | 1.02 | 1.01-1.03 | 0.002 | 0.7 | 0.69-0.71 | 0 |
| C10orf10 | 1 | 1.00-1.00 | 0.389 | NA | NA | NA |
| C10orf35 | 1.02 | 1.01-1.03 | 0.001 | 0.9 | 0.88-0.91 | 0 |
| C11orf21 | 0.92 | 0.7-1.21 | 0.533 | NA | NA | NA |
| C11orf65 | 1.12 | 0.66-1.88 | 0.674 | NA | NA | NA |
| C11orf68 | 1.01 | 1-1.02 | 0.003 | 1.08 | 1.07-1.09 | 0 |
| C11orf71 | 1 | 0.98-1.02 | 0.852 | NA | NA | NA |
| C12orf42 | 5.53 | 1.69-18.06 | 0.005 | 0.02 | 0-0.07 | 0 |
| C14orf93 | 1.11 | 1.06-1.17 | 0 | 0.46 | 0.43-0.49 | 0 |
| C16orf45 | 0.99 | 0.98-1.01 | 0.291 | NA | NA | NA |
| C16orf46 | 1.27 | 1.02-1.58 | 0.034 | 4.37 | 3.37-5.67 | 0 |
| C17orf96 | 1.01 | 0.99-1.03 | 0.456 | NA | NA | NA |
| C17orf97 | 0.99 | 0.91-1.07 | 0.726 | NA | NA | NA |
| C1orf194 | 1.36 | 0.93-1.98 | 0.108 | NA | NA | NA |
| C1orf198 | 1 | 1-1.01 | 0.318 | NA | NA | NA |
| C1orf228 | 1.04 | 0.96-1.13 | 0.341 | NA | NA | NA |
| C2CD4D | 1.08 | 0.86-1.35 | 0.497 | NA | NA | NA |
| C2orf66 | 2.97 | 1.28-6.9 | 0.011 | 0.04 | 0.01-0.14 | 0 |
| C2orf76 | 1.06 | 1.02-1.1 | 0.006 | 0.5 | 0.47-0.53 | 0 |
| C3orf14 | 1.11 | 1.04-1.18 | 0.002 | 0.57 | 0.53-0.62 | 0 |
| C7orf25 | 1.68 | 0.82-3.43 | 0.155 | NA | NA | NA |
| C7orf26 | 1.02 | 1.01-1.04 | 0.002 | 0.81 | 0.8-0.83 | 0 |
| CA9 | 1 | 1.00-1.00 | 0.128 | NA | NA | NA |
| CAPN10 | 1.18 | 1.11-1.25 | 0 | 0.41 | 0.38-0.44 | 0 |
| CARNS1 | 1.08 | 1.02-1.16 | 0.011 | 1.63 | 1.51-1.77 | 0 |
| CASD1 | 1.04 | 0.99-1.08 | 0.091 | NA | NA | NA |
| CATSPER3 | 1.12 | 0.93-1.35 | 0.232 | NA | NA | NA |
| CBX8 | 1.05 | 1.02-1.08 | 0.001 | 1.16 | 1.11-1.21 | 0 |
| CCDC102A | 1.03 | 1-1.06 | 0.054 | NA | NA | NA |
| CCDC103 | 1.09 | 0-22349.3 | 0.986 | NA | NA | NA |
| CCDC134 | 1.07 | 1.02-1.11 | 0.006 | 0.92 | 0.87-0.98 | 0.007 |
| CCDC138 | 1.16 | 1.03-1.31 | 0.013 | 0.13 | 0.11-0.15 | 0 |
| CCDC22 | 1.02 | 1-1.04 | 0.013 | 0.83 | 0.81-0.84 | 0 |
| CCDC86 | 1.01 | 1-1.02 | 0.002 | 1.25 | 1.23-1.26 | 0 |
| CCNYL1 | 1.04 | 1.01-1.06 | 0.004 | 0.49 | 0.47-0.5 | 0 |
| CD160 | 1.1 | 0.86-1.41 | 0.43 | NA | NA | NA |
| CD177 | 0.99 | 0.94-1.04 | 0.669 | NA | NA | NA |
| CD244 | 1.09 | 0.96-1.22 | 0.174 | NA | NA | NA |
| CD70 | 1.01 | 0.99-1.04 | 0.389 | NA | NA | NA |
| CDC23 | 1.04 | 1.02-1.06 | 0 | 1.48 | 1.45-1.52 | 0 |
| CDC34 | 1 | 1-1.01 | 0.042 | 0.97 | 0.97-0.98 | 0 |
| CDHR1 | 1 | 0.96-1.04 | 0.972 | NA | NA | NA |
| CDK5 | 1.02 | 1.01-1.04 | 0.01 | 0.88 | 0.86-0.89 | 0 |
| CDKL2 | 3.66 | 0.95-14.05 | 0.059 | NA | NA | NA |
| CELF6 | 1.95 | 0.18-21.5 | 0.585 | NA | NA | NA |
| CETN3 | 1.07 | 1.01-1.13 | 0.018 | 0.69 | 0.65-0.73 | 0 |
| CFAP44 | 1.14 | 1.04-1.24 | 0.003 | 0.28 | 0.24-0.32 | 0 |
| CH25H | 1 | 0.98-1.02 | 0.996 | NA | NA | NA |
| CHIC1 | 1.01 | 0.95-1.08 | 0.766 | NA | NA | NA |
| CHMP4B | 1 | 1.00-1.00 | 0.011 | 1.02 | 1.02-1.02 | 0 |
| CHMP6 | 1.01 | 1-1.02 | 0.013 | 1.08 | 1.07-1.09 | 0 |
| CHN1 | 1 | 0.97-1.02 | 0.811 | NA | NA | NA |
| CHPF | 1 | 1-1.01 | 0.008 | 1.02 | 1.02-1.02 | 0 |
| CHPF2 | 1.01 | 1-1.02 | 0.013 | 1.13 | 1.11-1.14 | 0 |
| CHTF8 | 1.01 | 1-1.02 | 0.157 | NA | NA | NA |
| CLASRP | 1.01 | 1-1.02 | 0.007 | 0.8 | 0.79-0.81 | 0 |
| CLCF1 | 1.03 | 1.01-1.05 | 0.01 | 0.73 | 0.71-0.75 | 0 |
| CLYBL | 0.97 | 0.94-0.99 | 0.013 | 1.08 | 1.05-1.11 | 0 |
| COG2 | 1.01 | 0.98-1.05 | 0.512 | NA | NA | NA |
| COL9A2 | 1.02 | 1.01-1.03 | 0.001 | 0.81 | 0.79-0.82 | 0 |
| COPG2 | 1.02 | 1-1.04 | 0.048 | 0.79 | 0.77-0.8 | 0 |
| CPE | 1 | 1.00-1.00 | 0.652 | NA | NA | NA |
| CPT1B | 1.14 | 1.03-1.26 | 0.012 | 0.76 | 0.67-0.86 | 0 |
| CRABP2 | 1 | 0.99-1.01 | 0.624 | NA | NA | NA |
| CRADD | 0.99 | 0.95-1.03 | 0.54 | NA | NA | NA |
| CREB3L4 | 1.02 | 1-1.03 | 0.025 | 1.25 | 1.23-1.28 | 0 |
| CRLS1 | 1 | 0.99-1 | 0.944 | NA | NA | NA |
| CROT | 0.99 | 0.98-1 | 0.233 | NA | NA | NA |
| CST1 | 1 | 1.00-1.00 | 0.41 | NA | NA | NA |
| CST2 | 1.03 | 0.98-1.07 | 0.23 | NA | NA | NA |
| CST4 | 1 | 0.97-1.03 | 0.865 | NA | NA | NA |
| CSTF3 | 1.05 | 1.02-1.08 | 0.001 | 0.61 | 0.59-0.64 | 0 |
| CTD.2568A17.1 | 1.48 | 0.82-2.65 | 0.191 | NA | NA | NA |
| CTNS | 1.01 | 0.99-1.04 | 0.304 | NA | NA | NA |
| CTPS1 | 1.01 | 1-1.02 | 0.241 | NA | NA | NA |
| CTSF | 1 | 1.00-1.00 | 0.985 | NA | NA | NA |
| CTSG | 0.97 | 0.94-1.01 | 0.167 | NA | NA | NA |
| CTSO | 1 | 0.99-1 | 0.105 | NA | NA | NA |
| CXorf65 | 0.93 | 0.68-1.26 | 0.632 | NA | NA | NA |
| CXXC1 | 1.03 | 1.01-1.04 | 0 | 1.74 | 1.7-1.77 | 0 |
| CYP4F22 | 1 | 1-1.01 | 0.316 | NA | NA | NA |
| DAPK3 | 1.01 | 1-1.01 | 0.215 | NA | NA | NA |
| DCAF4L1 | 1.09 | 0.88-1.37 | 0.43 | NA | NA | NA |
| DDX43 | 1.13 | 0.98-1.29 | 0.083 | NA | NA | NA |
| DGCR14 | 1.08 | 1.04-1.13 | 0 | 1.1 | 1.04-1.16 | 0.001 |
| DGKI | 1.35 | 0.95-1.9 | 0.09 | NA | NA | NA |
| DGKQ | 1.02 | 0.99-1.05 | 0.116 | NA | NA | NA |
| DIRAS3 | 0.98 | 0.94-1.03 | 0.477 | NA | NA | NA |
| DLGAP1 | 1.23 | 0.92-1.64 | 0.158 | NA | NA | NA |
| DMXL1 | 1.04 | 0.98-1.12 | 0.201 | NA | NA | NA |
| DNAL4 | 1.01 | 1-1.03 | 0.056 | NA | NA | NA |
| DNER | 1 | 0.99-1.01 | 0.612 | NA | NA | NA |
| DOLK | 1.02 | 1.01-1.03 | 0.004 | 0.93 | 0.91-0.94 | 0 |
| DPEP2 | 0.98 | 0.85-1.12 | 0.745 | NA | NA | NA |
| DROSHA | 1.03 | 1.02-1.05 | 0 | 0.77 | 0.76-0.79 | 0 |
| DTX3 | 1.02 | 1-1.04 | 0.137 | NA | NA | NA |
| DYNC2H1 | 1.53 | 1.19-1.96 | 0.001 | 0.14 | 0.11-0.18 | 0 |
| EDC3 | 1.08 | 1.05-1.11 | 0 | 1.28 | 1.24-1.32 | 0 |
| EEPD1 | 1.01 | 1-1.03 | 0.086 | NA | NA | NA |
| EFCAB13 | 1.25 | 0.99-1.57 | 0.063 | NA | NA | NA |
| EFCAB7 | 1.2 | 1.06-1.35 | 0.003 | 0.04 | 0.04-0.05 | 0 |
| EFNA5 | 1.02 | 1-1.04 | 0.073 | NA | NA | NA |
| EGR2 | 1 | 0.95-1.04 | 0.899 | NA | NA | NA |
| EGR3 | 1.01 | 0.89-1.14 | 0.917 | NA | NA | NA |
| EID3 | 1.28 | 1.18-1.39 | 0 | 1.65 | 1.49-1.81 | 0 |
| ELK1 | 1.02 | 1.01-1.03 | 0.002 | 1.18 | 1.16-1.2 | 0 |
| ELMOD2 | 1.03 | 1-1.07 | 0.046 | 0.78 | 0.74-0.81 | 0 |
| ENC1 | 1.01 | 1-1.01 | 0.033 | 1.21 | 1.2-1.23 | 0 |
| ENPP5 | 1.02 | 0.98-1.06 | 0.297 | NA | NA | NA |
| EPHB3 | 1.01 | 1-1.03 | 0.033 | 1.38 | 1.35-1.42 | 0 |
| EPHX2 | 1 | 0.99-1 | 0.164 | NA | NA | NA |
| EPM2A | 1.05 | 0.93-1.19 | 0.436 | NA | NA | NA |
| ERCC6 | 1.51 | 1.2-1.89 | 0 | 0.19 | 0.14-0.25 | 0 |
| ERCC6L | 1.15 | 1.06-1.25 | 0.001 | 2.49 | 2.13-2.92 | 0 |
| ERI3 | 1.01 | 1-1.01 | 0.001 | 1.06 | 1.05-1.07 | 0 |
| ERP27 | 1 | 1-1.01 | 0.216 | NA | NA | NA |
| ETV1 | 1.06 | 1.02-1.09 | 0.002 | 0.7 | 0.66-0.74 | 0 |
| ETV7 | 1.01 | 0.99-1.04 | 0.406 | NA | NA | NA |
| EVA1B | 1 | 0.99-1.01 | 0.73 | NA | NA | NA |
| EXTL2 | 1.04 | 1.01-1.07 | 0.011 | 1.1 | 1.05-1.15 | 0 |
| F8A1 | 1.02 | 1-1.03 | 0.009 | 0.93 | 0.92-0.95 | 0 |
| F8A3 | 2.92 | 0.57-14.98 | 0.2 | NA | NA | NA |
| FAM104B | 1.07 | 1.03-1.11 | 0 | 1.33 | 1.27-1.4 | 0 |
| FAM120AOS | 1.03 | 1.01-1.05 | 0.003 | 0.75 | 0.74-0.77 | 0 |
| FAM122A | 1.02 | 0.98-1.07 | 0.284 | NA | NA | NA |
| FAM13A | 0.99 | 0.95-1.02 | 0.441 | NA | NA | NA |
| FAM167A | 1 | 0.99-1.03 | 0.629 | NA | NA | NA |
| FAM171A1 | 1 | 1.00-1.00 | 0.448 | NA | NA | NA |
| FAM173A | 1.01 | 0.99-1.03 | 0.272 | NA | NA | NA |
| FAM175B | 1.03 | 1-1.06 | 0.038 | 0.9 | 0.87-0.93 | 0 |
| FAM184A | 1.07 | 1-1.14 | 0.053 | NA | NA | NA |
| FAM200A | 1.07 | 1-1.13 | 0.039 | 0.53 | 0.5-0.57 | 0 |
| FAM209B | 1.54 | 1.02-2.32 | 0.04 | 4.04 | 2.47-6.61 | 0 |
| FAM218A | 2.04 | 1.16-3.59 | 0.013 | 0 | 0-0 | 0 |
| FAM24B | 1.02 | 1-1.03 | 0.034 | 0.23 | 0.22-0.23 | 0 |
| FAM89B | 1.02 | 1.01-1.03 | 0 | 0.77 | 0.76-0.78 | 0 |
| FAN1 | 1.01 | 0.97-1.06 | 0.524 | NA | NA | NA |
| FASTKD5 | 1.02 | 1-1.05 | 0.055 | NA | NA | NA |
| FBXL22 | 1.6 | 1.01-2.53 | 0.043 | 1255.65 | 709.54-2222.11 | 0 |
| FBXO18 | 1.02 | 1.01-1.04 | 0.005 | 0.45 | 0.44-0.46 | 0 |
| FBXO2 | 1 | 1.00-1.00 | 0.886 | NA | NA | NA |
| FBXO41 | 1.17 | 1.05-1.31 | 0.004 | 1.51 | 1.27-1.8 | 0 |
| FBXO46 | 1.01 | 1-1.01 | 0.009 | 1.17 | 1.16-1.18 | 0 |
| FCRL3 | 1.05 | 1-1.11 | 0.058 | NA | NA | NA |
| FCRL6 | 1.02 | 0.89-1.16 | 0.804 | NA | NA | NA |
| FDXR | 1 | 0.99-1.01 | 0.713 | NA | NA | NA |
| FGF7 | 1.02 | 0.88-1.18 | 0.804 | NA | NA | NA |
| FGFRL1 | 1 | 0.99-1.01 | 0.973 | NA | NA | NA |
| FIZ1 | 1.1 | 1.03-1.16 | 0.002 | 0.43 | 0.4-0.47 | 0 |
| FKBPL | 1.02 | 1-1.04 | 0.021 | 1.06 | 1.04-1.09 | 0 |
| FOXJ2 | 1.07 | 1.03-1.12 | 0.002 | 0.5 | 0.47-0.52 | 0 |
| FRMD3 | 0.99 | 0.97-1.02 | 0.511 | NA | NA | NA |
| FSIP1 | 1.14 | 0.97-1.35 | 0.119 | NA | NA | NA |
| FUT10 | 1.13 | 1.04-1.22 | 0.002 | 0.16 | 0.14-0.18 | 0 |
| GADD45G | 1 | 1.00-1.00 | 0.963 | NA | NA | NA |
| GAR1 | 1.03 | 1.02-1.05 | 0 | 1.22 | 1.19-1.25 | 0 |
| GATA2 | 0.9 | 0.79-1.03 | 0.117 | NA | NA | NA |
| GFER | 1.01 | 1-1.02 | 0.145 | NA | NA | NA |
| GFI1 | 1.01 | 0.94-1.07 | 0.86 | NA | NA | NA |
| GHDC | 1.02 | 1.01-1.04 | 0 | 0.96 | 0.94-0.97 | 0 |
| GIN1 | 1.14 | 0.97-1.34 | 0.118 | NA | NA | NA |
| GLI4 | 1.01 | 0.99-1.03 | 0.18 | NA | NA | NA |
| GLYCTK | 1 | 1.00-1.00 | 0.686 | NA | NA | NA |
| GMPPA | 1.02 | 1-1.03 | 0.007 | 0.83 | 0.82-0.84 | 0 |
| GNG8 | 0.97 | 0.83-1.12 | 0.663 | NA | NA | NA |
| GPAM | 1 | 1.00-1.00 | 0.742 | NA | NA | NA |
| GPATCH3 | 1.02 | 1.01-1.04 | 0.003 | 1.16 | 1.14-1.18 | 0 |
| GPD2 | 1.08 | 1.04-1.12 | 0 | 1.03 | 0.98-1.09 | 0.262 |
| GPR146 | 0.97 | 0.86-1.09 | 0.594 | NA | NA | NA |
| GPR55 | 0.91 | 0.58-1.42 | 0.673 | NA | NA | NA |
| GPRASP1 | 1.1 | 1-1.22 | 0.055 | NA | NA | NA |
| GSTA4 | 1.01 | 1-1.01 | 0.007 | 0.94 | 0.93-0.94 | 0 |
| GZF1 | 1.08 | 1.02-1.14 | 0.008 | 1.17 | 1.1-1.25 | 0 |
| HBA1 | 0.83 | 0.64-1.06 | 0.138 | NA | NA | NA |
| HBA2 | 1 | 0.99-1 | 0.291 | NA | NA | NA |
| HBB | 1 | 0.99-1 | 0.321 | NA | NA | NA |
| HCRT | 4.3 | 1.78-10.42 | 0.001 | 0.01 | 0-0.03 | 0 |
| HEATR9 | 0.54 | 0.12-2.38 | 0.42 | NA | NA | NA |
| HHAT | 1.05 | 1.01-1.09 | 0.017 | 0.83 | 0.8-0.85 | 0 |
| HIBADH | 1 | 1.00-1.00 | 0.37 | NA | NA | NA |
| HIRA | 1.04 | 1-1.07 | 0.038 | 0.54 | 0.52-0.55 | 0 |
| HIST1H2AB | 1.18 | 0.79-1.76 | 0.427 | NA | NA | NA |
| HKDC1 | 1 | 1-1.01 | 0.591 | NA | NA | NA |
| HLF | 1 | 0.99-1 | 0.271 | NA | NA | NA |
| HN1L | 1.01 | 1-1.01 | 0.008 | 0.94 | 0.93-0.94 | 0 |
| HPS6 | 1.04 | 1.01-1.06 | 0.009 | 2.05 | 1.99-2.11 | 0 |
| HSD11B1 | 1 | 1.00-1.00 | 0.146 | NA | NA | NA |
| HSDL2 | 1 | 1.00-1.00 | 0.905 | NA | NA | NA |
| HTR2B | 0.98 | 0.96-1 | 0.066 | NA | NA | NA |
| HTRA1 | 1 | 1.00-1.00 | 0.348 | NA | NA | NA |
| ICA1 | 1 | 0.98-1.02 | 0.972 | NA | NA | NA |
| IDH1 | 1 | 1.00-1.00 | 0.322 | NA | NA | NA |
| IDUA | 1.01 | 0.99-1.03 | 0.173 | NA | NA | NA |
| IFITM10 | 1.01 | 1-1.02 | 0.025 | 1.01 | 0.99-1.02 | 0.289 |
| IFT43 | 1.02 | 0.99-1.06 | 0.161 | NA | NA | NA |
| IL11RA | 1.02 | 1-1.04 | 0.075 | NA | NA | NA |
| IL12A | 1.64 | 1.25-2.16 | 0 | 0.04 | 0.03-0.06 | 0 |
| IL15RA | 1.02 | 1.01-1.03 | 0 | 1.07 | 1.06-1.09 | 0 |
| IL17A | 0.36 | 0.01-12.1 | 0.57 | NA | NA | NA |
| IL17D | 1.02 | 1-1.04 | 0.034 | 1.15 | 1.11-1.18 | 0 |
| IL17F | 0.46 | 0.14-1.53 | 0.207 | NA | NA | NA |
| IL1RL1 | 0.87 | 0.73-1.05 | 0.147 | NA | NA | NA |
| IL26 | 1.63 | 0.54-4.87 | 0.385 | NA | NA | NA |
| IL9R | 1.53 | 0.47-4.96 | 0.483 | NA | NA | NA |
| ILVBL | 1 | 0.99-1 | 0.273 | NA | NA | NA |
| IMPACT | 1.02 | 0.99-1.04 | 0.183 | NA | NA | NA |
| INPP5A | 1.01 | 0.99-1.03 | 0.327 | NA | NA | NA |
| INVS | 1.12 | 1.05-1.2 | 0.001 | 4.05 | 3.75-4.38 | 0 |
| JAM3 | 1 | 0.97-1.03 | 0.966 | NA | NA | NA |
| JOSD2 | 1 | 1-1.01 | 0.457 | NA | NA | NA |
| JRK | 1.12 | 1.03-1.22 | 0.008 | 0.03 | 0.03-0.03 | 0 |
| KBTBD7 | 1.04 | 0.97-1.11 | 0.224 | NA | NA | NA |
| KCND1 | 1.09 | 0.9-1.31 | 0.377 | NA | NA | NA |
| KCNJ13 | 1.16 | 0.94-1.42 | 0.158 | NA | NA | NA |
| KCNN4 | 1.02 | 0.95-1.1 | 0.608 | NA | NA | NA |
| KCTD1 | 1.06 | 0.99-1.14 | 0.113 | NA | NA | NA |
| KCTD11 | 1.03 | 0.99-1.06 | 0.123 | NA | NA | NA |
| KCTD16 | 1.38 | 0.21-9.25 | 0.738 | NA | NA | NA |
| KCTD17 | 1.01 | 1-1.01 | 0.001 | 0.99 | 0.98-1 | 0.02 |
| KIAA0895L | 1.06 | 1.01-1.11 | 0.015 | 2.91 | 2.73-3.11 | 0 |
| KIAA2013 | 1 | 1-1.01 | 0.219 | NA | NA | NA |
| KIR2DL3 | 0.62 | 0.17-2.27 | 0.469 | NA | NA | NA |
| KIZ | 1.01 | 1-1.03 | 0.029 | 1.06 | 1.04-1.08 | 0 |
| KLHDC10 | 1 | 0.99-1.01 | 0.995 | NA | NA | NA |
| KLHL29 | 1.04 | 1-1.08 | 0.061 | NA | NA | NA |
| KLHL3 | 0.99 | 0.88-1.12 | 0.889 | NA | NA | NA |
| KLRG1 | 1.12 | 0.98-1.29 | 0.109 | NA | NA | NA |
| KRCC1 | 1.01 | 1-1.02 | 0.02 | 1.02 | 1.01-1.03 | 0 |
| KRT19 | 1 | 1.00-1.00 | 0.109 | NA | NA | NA |
| KRT23 | 1 | 1.00-1.00 | 0.002 | 1.02 | 1.01-1.02 | 0 |
| KRTCAP3 | 1 | 1-1.01 | 0.761 | NA | NA | NA |
| L3HYPDH | 1.02 | 0.99-1.05 | 0.162 | NA | NA | NA |
| LACC1 | 1.03 | 0.96-1.1 | 0.378 | NA | NA | NA |
| LAMB1 | 1.01 | 1-1.01 | 0 | 1 | 1-1.01 | 0.155 |
| LAYN | 1.01 | 0.99-1.04 | 0.362 | NA | NA | NA |
| LCAT | 0.99 | 0.99-1 | 0.002 | 1 | 1.00-1.00 | 0.094 |
| LCN10 | 0.41 | 0.03-6.47 | 0.527 | NA | NA | NA |
| LDB1 | 1.02 | 1.01-1.03 | 0.001 | 0.74 | 0.73-0.75 | 0 |
| LGR4 | 1 | 1-1.01 | 0.835 | NA | NA | NA |
| LIN7A | 0.99 | 0.97-1.01 | 0.339 | NA | NA | NA |
| LINGO3 | 1.57 | 1-2.46 | 0.05 | NA | NA | NA |
| LMCD1 | 1.01 | 0.98-1.04 | 0.427 | NA | NA | NA |
| LMF1 | 0.98 | 0.94-1.03 | 0.483 | NA | NA | NA |
| LOX | 1.01 | 0.99-1.03 | 0.256 | NA | NA | NA |
| LPL | 1.02 | 1-1.05 | 0.044 | 1.08 | 1.05-1.1 | 0 |
| LRCH1 | 1 | 0.95-1.05 | 0.922 | NA | NA | NA |
| LRFN1 | 1.06 | 1.01-1.11 | 0.013 | 1.06 | 0.99-1.13 | 0.076 |
| LRRC29 | 1.03 | 0.9-1.16 | 0.698 | NA | NA | NA |
| LRRC46 | 1.28 | 1.04-1.56 | 0.017 | 4.36 | 3.39-5.62 | 0 |
| LRRC61 | 1.01 | 1-1.01 | 0.038 | 0.99 | 0.98-1 | 0.012 |
| LRRCC1 | 1.04 | 1-1.08 | 0.031 | 0.54 | 0.51-0.56 | 0 |
| LUZP6 | NA | NA-NA | NA | NA | NA | NA |
| LYG1 | 1.1 | 1-1.2 | 0.043 | 0.1 | 0.09-0.11 | 0 |
| LYG2 | 1.22 | 1.08-1.38 | 0.002 | 0.68 | 0.58-0.79 | 0 |
| LYPLAL1 | 1 | 0.99-1.01 | 0.346 | NA | NA | NA |
| LYRM9 | 1.01 | 0.91-1.12 | 0.818 | NA | NA | NA |
| MAGEF1 | 1 | 1-1.01 | 0.132 | NA | NA | NA |
| MANBAL | 1.01 | 1-1.01 | 0.004 | 1.14 | 1.13-1.14 | 0 |
| MAP2K5 | 1.05 | 0.99-1.1 | 0.084 | NA | NA | NA |
| MAP3K5 | 1 | 0.98-1.02 | 0.737 | NA | NA | NA |
| MB21D2 | 1.06 | 0.97-1.16 | 0.202 | NA | NA | NA |
| MBD1 | 1.03 | 1.01-1.05 | 0.005 | 0.72 | 0.7-0.74 | 0 |
| MCAT | 1.01 | 0.99-1.03 | 0.225 | NA | NA | NA |
| MDC1 | 1.02 | 1-1.03 | 0.065 | NA | NA | NA |
| MDP1 | 1.14 | 1.02-1.28 | 0.02 | 0.15 | 0.13-0.17 | 0 |
| MEIOB | 1.44 | 1.13-1.84 | 0.003 | 42.15 | 27.28-65.11 | 0 |
| MEN1 | 1.03 | 1.01-1.04 | 0.002 | 1.04 | 1.02-1.06 | 0.001 |
| MEST | 1 | 1.00-1.00 | 0.161 | NA | NA | NA |
| MEX3B | 1.14 | 1-1.3 | 0.049 | 0.81 | 0.71-0.92 | 0.001 |
| MFSD6 | 1.02 | 1-1.04 | 0.087 | NA | NA | NA |
| MINA | 1.04 | 1.01-1.07 | 0.013 | 0.96 | 0.92-0.99 | 0.012 |
| MIPEP | 1.01 | 0.98-1.03 | 0.664 | NA | NA | NA |
| MLH3 | 1.06 | 1.01-1.11 | 0.013 | 1.26 | 1.19-1.34 | 0 |
| MLLT1 | 1.01 | 1-1.02 | 0.019 | 0.95 | 0.94-0.96 | 0 |
| MMAA | 0.94 | 0.89-0.99 | 0.031 | 0.49 | 0.46-0.52 | 0 |
| MMEL1 | 1.29 | 1.01-1.66 | 0.045 | 18.67 | 14.2-24.55 | 0 |
| MMGT1 | 1.02 | 1-1.04 | 0.081 | NA | NA | NA |
| MMP23B | 0.74 | 0.27-2.06 | 0.57 | NA | NA | NA |
| MOGS | 1.01 | 1.01-1.02 | 0 | 1.05 | 1.04-1.06 | 0 |
| MOSPD1 | 1 | 0.99-1.01 | 0.935 | NA | NA | NA |
| MPZ | 1 | 1-1.01 | 0.009 | 1.02 | 1.01-1.02 | 0 |
| MRGBP | 1.03 | 1.02-1.04 | 0 | 0.7 | 0.68-0.71 | 0 |
| MRPS2 | 1 | 0.99-1.01 | 0.712 | NA | NA | NA |
| MSRB2 | 1 | 1-1.01 | 0.045 | 1.04 | 1.03-1.04 | 0 |
| MTMR10 | 1 | 0.97-1.04 | 0.82 | NA | NA | NA |
| MVB12B | 1.05 | 0.99-1.1 | 0.079 | NA | NA | NA |
| MYO1D | 1 | 0.99-1.01 | 0.959 | NA | NA | NA |
| MYPOP | 1.02 | 1-1.05 | 0.069 | NA | NA | NA |
| MYRF | 1 | 1-1.01 | 0.05 | NA | NA | NA |
| MYRIP | 0.99 | 0.98-1 | 0.076 | NA | NA | NA |
| MZF1 | 1.03 | 0.99-1.07 | 0.096 | NA | NA | NA |
| N4BP3 | 1.24 | 1.09-1.43 | 0.002 | 5.95 | 5-7.08 | 0 |
| NAALAD2 | 1.1 | 0.99-1.23 | 0.071 | NA | NA | NA |
| NAALADL1 | 1 | 0.99-1.01 | 0.471 | NA | NA | NA |
| NAB2 | 1.01 | 1-1.03 | 0.055 | NA | NA | NA |
| NCKIPSD | 1.03 | 1.01-1.04 | 0 | 1.12 | 1.1-1.13 | 0 |
| NDFIP2 | 1 | 0.99-1.02 | 0.808 | NA | NA | NA |
| NEBL | 1.02 | 0.98-1.07 | 0.278 | NA | NA | NA |
| NECAB3 | 1.01 | 1-1.01 | 0.072 | NA | NA | NA |
| NELFB | 1.01 | 1-1.01 | 0.005 | 0.94 | 0.93-0.94 | 0 |
| NENF | 1 | 1.00-1.00 | 0.017 | 0.98 | 0.98-0.98 | 0 |
| NEURL1 | 1.04 | 0.99-1.09 | 0.165 | NA | NA | NA |
| NFIL3 | 1 | 1.00-1.00 | 0.483 | NA | NA | NA |
| NFKBIL1 | 1.01 | 1-1.01 | 0.06 | NA | NA | NA |
| NHEJ1 | 1.18 | 0.91-1.53 | 0.209 | NA | NA | NA |
| NID2 | 1.02 | 0.99-1.05 | 0.262 | NA | NA | NA |
| NKX3.1 | 1.05 | 0.95-1.16 | 0.37 | NA | NA | NA |
| NMU | 1.03 | 0.94-1.14 | 0.531 | NA | NA | NA |
| NOTUM | 1 | 1.00-1.00 | 0.819 | NA | NA | NA |
| NPHP4 | 1.11 | 0.98-1.26 | 0.108 | NA | NA | NA |
| NPM2 | 0.98 | 0.97-1 | 0.137 | NA | NA | NA |
| NPPA | 0.95 | 0.7-1.29 | 0.743 | NA | NA | NA |
| NPPC | 1.17 | 0.85-1.59 | 0.331 | NA | NA | NA |
| NPW | 1 | 1.00-1.00 | 0.816 | NA | NA | NA |
| NR3C2 | 1.04 | 0.97-1.1 | 0.252 | NA | NA | NA |
| NSMAF | 1.04 | 1.01-1.08 | 0.022 | 1.35 | 1.28-1.41 | 0 |
| NSMF | 1.01 | 1-1.02 | 0.019 | 1.05 | 1.03-1.06 | 0 |
| NUDT13 | 0.98 | 0.94-1.03 | 0.441 | NA | NA | NA |
| OGFR | 1.01 | 1-1.01 | 0.134 | NA | NA | NA |
| OOEP | 1.21 | 1.06-1.37 | 0.004 | 1.17 | 0.99-1.39 | 0.071 |
| ORMDL3 | 1 | 1.00-1.00 | 0.763 | NA | NA | NA |
| OSBPL7 | 1.09 | 1.01-1.18 | 0.032 | 0.86 | 0.78-0.95 | 0.003 |
| OSGEPL1 | 1.1 | 1.03-1.16 | 0.002 | 0.85 | 0.79-0.91 | 0 |
| OSGIN2 | 1.02 | 1-1.04 | 0.042 | 1.12 | 1.1-1.14 | 0 |
| OXLD1 | 1.02 | 1.01-1.03 | 0 | 1.3 | 1.29-1.31 | 0 |
| P2RY14 | 0.89 | 0.68-1.16 | 0.387 | NA | NA | NA |
| PACRGL | 1.09 | 0.99-1.2 | 0.088 | NA | NA | NA |
| PAIP2B | 1 | 0.98-1.03 | 0.859 | NA | NA | NA |
| PAM16 | 1.03 | 0.99-1.06 | 0.118 | NA | NA | NA |
| PARD6A | 1.01 | 1-1.03 | 0.085 | NA | NA | NA |
| PARP12 | 1.02 | 1-1.03 | 0.034 | 1.19 | 1.17-1.21 | 0 |
| PARP2 | 1.05 | 1.03-1.08 | 0 | 1.35 | 1.31-1.39 | 0 |
| PCDHGA10 | 0.95 | 0.85-1.07 | 0.406 | NA | NA | NA |
| PCED1A | 1.01 | 1-1.02 | 0.001 | 0.99 | 0.98-1 | 0.029 |
| PCSK1N | 1 | 1-1.01 | 0.114 | NA | NA | NA |
| PCYT2 | 1 | 1-1.01 | 0.62 | NA | NA | NA |
| PDCD1LG2 | 0.99 | 0.96-1.03 | 0.672 | NA | NA | NA |
| PDF | 1.02 | 0.97-1.07 | 0.485 | NA | NA | NA |
| PDLIM7 | 1.01 | 1-1.02 | 0.012 | 0.71 | 0.7-0.72 | 0 |
| PELI3 | 1.03 | 0.99-1.07 | 0.184 | NA | NA | NA |
| PEX6 | 1.01 | 1-1.01 | 0.018 | 0.99 | 0.99-1 | 0.004 |
| PFKP | 1 | 1.00-1.00 | 0.368 | NA | NA | NA |
| PGBD3 | 1.17 | 0.01-111.04 | 0.945 | NA | NA | NA |
| PGRMC2 | 1.01 | 1-1.02 | 0.07 | NA | NA | NA |
| PHF1 | 1.01 | 1-1.02 | 0.005 | 1.11 | 1.1-1.12 | 0 |
| PHF7 | 1.02 | 0.95-1.1 | 0.559 | NA | NA | NA |
| PHOSPHO2 | 1.19 | 1.1-1.29 | 0 | 5.09 | 4.66-5.56 | 0 |
| PI16 | 0.99 | 0.89-1.11 | 0.924 | NA | NA | NA |
| PI4K2B | 0.01 | 0-1.88 | 0.082 | NA | NA | NA |
| PIBF1 | 1.02 | 0.98-1.07 | 0.333 | NA | NA | NA |
| PIP5K1B | 1.03 | 0.96-1.1 | 0.379 | NA | NA | NA |
| PLAGL1 | 1.07 | 1-1.13 | 0.04 | 2.72 | 2.51-2.95 | 0 |
| PLB1 | 1.2 | 0.96-1.48 | 0.104 | NA | NA | NA |
| PLCB2 | 1.01 | 0.97-1.06 | 0.587 | NA | NA | NA |
| PLCH2 | 0.97 | 0.94-1 | 0.05 | NA | NA | NA |
| PLCL2 | 1.03 | 1-1.07 | 0.062 | NA | NA | NA |
| PLEKHA8 | 1.24 | 1.11-1.38 | 0 | 1.74 | 1.5-2.03 | 0 |
| PLEKHG4 | 1.02 | 0.99-1.05 | 0.201 | NA | NA | NA |
| PLEKHG7 | 1.09 | 0.65-1.83 | 0.736 | NA | NA | NA |
| PLXND1 | 1 | 1-1.01 | 0.45 | NA | NA | NA |
| PM20D1 | 0.73 | 0.39-1.37 | 0.323 | NA | NA | NA |
| PMCH | 1.46 | 0.54-3.98 | 0.457 | NA | NA | NA |
| POFUT2 | 1.04 | 1.02-1.07 | 0.001 | 2.41 | 2.33-2.5 | 0 |
| POLE2 | 1.08 | 1.04-1.11 | 0 | 1.1 | 1.06-1.15 | 0 |
| POLI | 1.11 | 1.02-1.2 | 0.012 | 0.04 | 0.04-0.05 | 0 |
| POLR3D | 1.01 | 1-1.02 | 0.123 | NA | NA | NA |
| POLR3G | 1.33 | 1.18-1.49 | 0 | 1.57 | 1.38-1.79 | 0 |
| POLRMT | 1.01 | 1-1.02 | 0.032 | 0.95 | 0.94-0.96 | 0 |
| PPM1M | 1.02 | 1-1.04 | 0.109 | NA | NA | NA |
| PPOX | 1.04 | 1.01-1.06 | 0.005 | 1.05 | 1.02-1.08 | 0.001 |
| PPP1R12C | 1.01 | 1-1.02 | 0.183 | NA | NA | NA |
| PPP1R21 | 1.01 | 0.99-1.03 | 0.211 | NA | NA | NA |
| PPP1R35 | 1.01 | 1-1.02 | 0.003 | 0.9 | 0.89-0.91 | 0 |
| PPP4R1 | 1.03 | 1-1.05 | 0.021 | 0.9 | 0.87-0.92 | 0 |
| PPT2 | 1.02 | 1-1.04 | 0.013 | 1.08 | 1.05-1.1 | 0 |
| PRDM11 | 1.19 | 0.98-1.44 | 0.078 | NA | NA | NA |
| PRH2 | 1 | 0.96-1.04 | 0.837 | NA | NA | NA |
| PRICKLE3 | 1.07 | 1-1.15 | 0.06 | NA | NA | NA |
| PRKAR1B | 1.01 | 0.99-1.03 | 0.157 | NA | NA | NA |
| PRKCD | 1.02 | 1-1.03 | 0.008 | 0.94 | 0.92-0.95 | 0 |
| PRKCZ | 1.01 | 0.95-1.07 | 0.703 | NA | NA | NA |
| PRMT6 | 1.01 | 0.99-1.03 | 0.228 | NA | NA | NA |
| PRR5L | 1 | 0.96-1.04 | 0.957 | NA | NA | NA |
| PRX | 1.09 | 1.02-1.17 | 0.017 | 2.88 | 2.6-3.2 | 0 |
| PTAR1 | 1.02 | 0.98-1.06 | 0.376 | NA | NA | NA |
| PTGES2 | 1.01 | 1-1.01 | 0.031 | 1.07 | 1.07-1.08 | 0 |
| PTPRM | 1.02 | 1-1.03 | 0.025 | 1.26 | 1.24-1.27 | 0 |
| PWWP2A | 1.17 | 1.09-1.26 | 0 | 0.51 | 0.46-0.56 | 0 |
| QSOX2 | 1.04 | 1.01-1.06 | 0.002 | 1.04 | 1.01-1.07 | 0.004 |
| RAB28 | 1.03 | 1-1.06 | 0.049 | 0.98 | 0.94-1.01 | 0.202 |
| RAB37 | 1.02 | 1-1.04 | 0.127 | NA | NA | NA |
| RAB39B | 0.87 | 0.62-1.21 | 0.4 | NA | NA | NA |
| RAB3A | 1.01 | 0.98-1.05 | 0.569 | NA | NA | NA |
| RAB43 | 1 | 0.91-1.11 | 0.947 | NA | NA | NA |
| RASL11B | 1.03 | 1-1.07 | 0.064 | NA | NA | NA |
| RBM12B | 1.16 | 1.08-1.26 | 0 | 13.48 | 12.33-14.73 | 0 |
| RCN3 | 1 | 0.99-1.02 | 0.484 | NA | NA | NA |
| RDM1 | 1.18 | 1.08-1.28 | 0 | 1.93 | 1.69-2.2 | 0 |
| RECQL4 | 1.01 | 1-1.01 | 0.001 | 1.14 | 1.13-1.16 | 0 |
| REP15 | 1.08 | 0.95-1.23 | 0.259 | NA | NA | NA |
| REPIN1 | 1 | 1-1.01 | 0.031 | 0.83 | 0.83-0.84 | 0 |
| RETSAT | 1 | 0.99-1 | 0.025 | 1 | 0.99-1 | 0.165 |
| RFESD | 1.65 | 1.05-2.6 | 0.029 | 0.23 | 0.14-0.36 | 0 |
| RFNG | 1.01 | 1-1.01 | 0.012 | 0.89 | 0.88-0.9 | 0 |
| RGL2 | 1 | 1-1.01 | 0.3 | NA | NA | NA |
| RGP1 | 1.03 | 1-1.05 | 0.033 | 3.36 | 3.26-3.48 | 0 |
| RGS13 | 0.49 | 0.21-1.12 | 0.09 | NA | NA | NA |
| RGS4 | 1.01 | 0.99-1.02 | 0.299 | NA | NA | NA |
| RHEBL1 | 1.17 | 1.08-1.27 | 0 | 0.86 | 0.76-0.97 | 0.017 |
| RHOT1 | 1.05 | 1.02-1.09 | 0.001 | 0.84 | 0.81-0.87 | 0 |
| RHOT2 | 1.02 | 1.01-1.03 | 0 | 0.78 | 0.77-0.79 | 0 |
| RIC8B | 1.02 | 0.99-1.05 | 0.115 | NA | NA | NA |
| RIMKLB | 1.03 | 0.91-1.16 | 0.659 | NA | NA | NA |
| RLN1 | 0.7 | 0.22-2.23 | 0.551 | NA | NA | NA |
| RNF121 | 1.02 | 1-1.05 | 0.05 | NA | NA | NA |
| RNF25 | 1.03 | 1.02-1.05 | 0 | 0.95 | 0.93-0.97 | 0 |
| RNF26 | 1.01 | 1-1.02 | 0.004 | 0.82 | 0.81-0.83 | 0 |
| RNLS | 1.01 | 0.98-1.04 | 0.681 | NA | NA | NA |
| RP11.195F19.5 | 1.43 | 1.19-1.72 | 0 | 2.28 | 1.81-2.87 | 0 |
| RP11.392E22.9 | 1.47 | 1.08-1.99 | 0.014 | 634758.4 | 410691.96-981071.53 | 0 |
| RP11.446E24.4 | 2.74 | 0.03-244.76 | 0.661 | NA | NA | NA |
| RP5.850E9.3 | 2.27 | 0.69-7.47 | 0.177 | NA | NA | NA |
| RPUSD1 | 1.02 | 1-1.03 | 0.049 | 0.91 | 0.89-0.93 | 0 |
| RSC1A1 | NA | NA-NA | NA | NA | NA | NA |
| RSG1 | 1.04 | 0.98-1.11 | 0.209 | NA | NA | NA |
| RTEL1 | 1.82 | 0.95-3.47 | 0.071 | NA | NA | NA |
| RUNDC3B | 0.99 | 0.96-1.02 | 0.475 | NA | NA | NA |
| SAP30L | 1.01 | 0.99-1.03 | 0.415 | NA | NA | NA |
| SASS6 | 1.1 | 1.03-1.16 | 0.002 | 0.55 | 0.5-0.59 | 0 |
| SBK1 | 1.11 | 1.04-1.17 | 0.001 | 3.65 | 3.29-4.06 | 0 |
| SCAF1 | 1.01 | 1-1.02 | 0.002 | 0.98 | 0.97-0.99 | 0 |
| SCAPER | 1.08 | 0.98-1.19 | 0.13 | NA | NA | NA |
| SCG5 | 1.01 | 0.99-1.03 | 0.162 | NA | NA | NA |
| SCML1 | 1.02 | 1-1.03 | 0.054 | NA | NA | NA |
| SCRIB | 1.01 | 1-1.01 | 0.04 | 1 | 0.99-1 | 0.211 |
| SEC61A2 | 1.06 | 1.02-1.11 | 0.004 | 1.46 | 1.38-1.54 | 0 |
| SECTM1 | 1 | 0.99-1.01 | 0.763 | NA | NA | NA |
| SENP1 | 1.11 | 1.05-1.17 | 0 | 0.02 | 0.02-0.03 | 0 |
| SEPHS1 | 1.02 | 1.01-1.03 | 0 | 1.3 | 1.29-1.32 | 0 |
| SERGEF | 1.04 | 1.02-1.07 | 0 | 0.99 | 0.96-1.01 | 0.316 |
| SERPINI1 | 1.01 | 1.01-1.02 | 0.001 | 0.89 | 0.88-0.9 | 0 |
| SGK223 | 1.01 | 0.99-1.02 | 0.55 | NA | NA | NA |
| SIRT3 | 1 | 0.97-1.04 | 0.802 | NA | NA | NA |
| SLC12A4 | 1 | 0.97-1.02 | 0.67 | NA | NA | NA |
| SLC35B3 | 1.01 | 1-1.02 | 0.205 | NA | NA | NA |
| SLC35G1 | 1.04 | 0.98-1.1 | 0.205 | NA | NA | NA |
| SLC39A7 | 1 | 1.00-1.00 | 0.139 | NA | NA | NA |
| SLC41A3 | 1.03 | 1.01-1.05 | 0.005 | 1.04 | 1.02-1.07 | 0.001 |
| SLC45A4 | 1.03 | 1.01-1.05 | 0.008 | 1.02 | 0.99-1.04 | 0.204 |
| SLC46A3 | 1 | 0.99-1 | 0.336 | NA | NA | NA |
| SLC6A8 | 1 | 1.00-1.00 | 0.919 | NA | NA | NA |
| SLFN14 | 0.36 | 0.02-8.45 | 0.527 | NA | NA | NA |
| SMARCD2 | 1.01 | 1-1.01 | 0.062 | NA | NA | NA |
| SMG8 | 1.05 | 1.02-1.08 | 0.003 | 1.01 | 0.97-1.04 | 0.701 |
| SMYD3 | NA | NA-NA | NA | NA | NA | NA |
| SNAI1 | 1 | 0.97-1.03 | 0.894 | NA | NA | NA |
| SNAI3 | 1.04 | 0.9-1.2 | 0.572 | NA | NA | NA |
| SOCS5 | 1.05 | 1.01-1.08 | 0.011 | 1.24 | 1.19-1.29 | 0 |
| SOX8 | 1.53 | 0.93-2.52 | 0.096 | NA | NA | NA |
| SPRED2 | 1.01 | 0.99-1.03 | 0.17 | NA | NA | NA |
| SPRYD3 | 1.01 | 1-1.02 | 0.051 | NA | NA | NA |
| SRF | 1.01 | 1-1.02 | 0.151 | NA | NA | NA |
| SRXN1 | 1.08 | 1.05-1.12 | 0 | 1.3 | 1.25-1.34 | 0 |
| SSC5D | 1.01 | 0.93-1.09 | 0.868 | NA | NA | NA |
| SSH3 | 1.01 | 1-1.02 | 0.206 | NA | NA | NA |
| ST7 | 1 | 0.97-1.03 | 0.952 | NA | NA | NA |
| STX10 | 1.01 | 1-1.01 | 0.001 | 1.06 | 1.05-1.06 | 0 |
| STX1A | 1.04 | 1-1.07 | 0.032 | 1.29 | 1.2-1.39 | 0 |
| STYK1 | 1.1 | 0.85-1.42 | 0.455 | NA | NA | NA |
| SWI5 | 1.01 | 1-1.01 | 0.141 | NA | NA | NA |
| SYNGR3 | 0.98 | 0.94-1.03 | 0.496 | NA | NA | NA |
| TAF15 | 1.01 | 1-1.02 | 0.004 | 0.91 | 0.91-0.92 | 0 |
| TAS2R30 | 0.38 | 0-124.26 | 0.741 | NA | NA | NA |
| TAS2R31 | 1.64 | 0.51-5.23 | 0.407 | NA | NA | NA |
| TAS2R43 | 2.53 | 0.17-36.91 | 0.498 | NA | NA | NA |
| TAS2R46 | 7.44 | 0.31-181.26 | 0.218 | NA | NA | NA |
| TAX1BP3 | 1 | 1-1.01 | 0.116 | NA | NA | NA |
| TBC1D13 | 1.02 | 1.01-1.03 | 0.002 | 1.13 | 1.11-1.14 | 0 |
| TBC1D3L | 1.18 | 1.01-1.38 | 0.038 | 0.1 | 0.08-0.13 | 0 |
| TBCEL | 1.01 | 0.98-1.05 | 0.435 | NA | NA | NA |
| TCP11L2 | 1.08 | 0.91-1.29 | 0.381 | NA | NA | NA |
| TELO2 | 1.03 | 1.01-1.04 | 0.002 | 0.82 | 0.81-0.84 | 0 |
| TESK2 | 1.02 | 0.97-1.07 | 0.422 | NA | NA | NA |
| TFAP2E | 1.02 | 0.92-1.14 | 0.681 | NA | NA | NA |
| TFCP2 | 1.03 | 1.01-1.05 | 0.002 | 1.77 | 1.73-1.82 | 0 |
| TGDS | 1 | 0.99-1.01 | 0.991 | NA | NA | NA |
| THAP11 | 1.02 | 1.01-1.03 | 0.006 | 1.34 | 1.32-1.37 | 0 |
| THBS3 | 1.02 | 1-1.04 | 0.086 | NA | NA | NA |
| THEM6 | 1 | 1.00-1.00 | 0.932 | NA | NA | NA |
| THSD1 | 0.98 | 0.95-1.01 | 0.218 | NA | NA | NA |
| TICAM2 | 6.05 | 0.58-63.45 | 0.133 | NA | NA | NA |
| TIMM9 | 1.02 | 1.01-1.03 | 0 | 1.08 | 1.07-1.1 | 0 |
| TLCD1 | 1.01 | 1-1.01 | 0.009 | 0.94 | 0.93-0.95 | 0 |
| TMEM129 | 1 | 0.99-1.01 | 0.813 | NA | NA | NA |
| TMEM155 | 0.97 | 0.84-1.11 | 0.649 | NA | NA | NA |
| TMEM185B | 1.03 | 1.01-1.04 | 0.001 | 0.99 | 0.97-1.01 | 0.259 |
| TMEM199 | 1.07 | 1.03-1.11 | 0.001 | 1.18 | 1.12-1.24 | 0 |
| TMEM203 | 1.01 | 1-1.01 | 0.017 | 0.91 | 0.9-0.92 | 0 |
| TMEM25 | 1 | 0.97-1.04 | 0.911 | NA | NA | NA |
| TMEM251 | 1.03 | 1.01-1.05 | 0 | 0.74 | 0.73-0.76 | 0 |
| TMEM69 | 1.03 | 1.01-1.04 | 0 | 0.94 | 0.93-0.96 | 0 |
| TMPPE | 1.25 | 0.93-1.67 | 0.138 | NA | NA | NA |
| TMPRSS4.AS1 | 0.62 | 0.04-9.16 | 0.729 | NA | NA | NA |
| TMPRSS6 | 1 | 1.00-1.00 | 0.248 | NA | NA | NA |
| TMTC2 | 1.12 | 0.98-1.27 | 0.089 | NA | NA | NA |
| TNFRSF13B | 1.12 | 0.85-1.46 | 0.422 | NA | NA | NA |
| TNFRSF4 | 1.01 | 0.98-1.04 | 0.36 | NA | NA | NA |
| TNFRSF8 | 1.07 | 0.78-1.47 | 0.68 | NA | NA | NA |
| TNFSF4 | 1.01 | 0.99-1.04 | 0.268 | NA | NA | NA |
| TOP3B | 1.38 | 0.35-5.39 | 0.642 | NA | NA | NA |
| TP53I11 | 1.01 | 0.99-1.02 | 0.267 | NA | NA | NA |
| TP53I13 | 1.01 | 1-1.02 | 0.03 | 1.1 | 1.09-1.11 | 0 |
| TPRG1L | 1 | 1.00-1.00 | 0.771 | NA | NA | NA |
| TRADD | 1.02 | 1-1.03 | 0.055 | NA | NA | NA |
| TRIM25 | 1.02 | 1-1.04 | 0.043 | 0.98 | 0.96-1 | 0.046 |
| TRIM62 | 1.03 | 0.97-1.08 | 0.354 | NA | NA | NA |
| TRMT10A | 1.04 | 0.95-1.13 | 0.398 | NA | NA | NA |
| TRMT2A | 1.02 | 1.01-1.03 | 0.006 | 1.08 | 1.07-1.1 | 0 |
| TRMT61B | 1.03 | 0.99-1.06 | 0.106 | NA | NA | NA |
| TRPT1 | 1.01 | 1-1.02 | 0.239 | NA | NA | NA |
| TSHZ1 | 1.05 | 1.01-1.1 | 0.018 | 0.68 | 0.65-0.71 | 0 |
| TSPAN32 | 0.88 | 0.68-1.16 | 0.37 | NA | NA | NA |
| TSTD3 | 1.25 | 1.1-1.42 | 0 | 0.72 | 0.62-0.85 | 0 |
| TTC12 | 1.08 | 0.99-1.17 | 0.086 | NA | NA | NA |
| TTI1 | 1.04 | 1.02-1.06 | 0 | 1.38 | 1.35-1.42 | 0 |
| TWIST1 | 1.01 | 0.99-1.03 | 0.529 | NA | NA | NA |
| TXLNB | 1.05 | 0.92-1.21 | 0.469 | NA | NA | NA |
| UCKL1 | 1.01 | 1.01-1.02 | 0.001 | 1.05 | 1.04-1.06 | 0 |
| UGT3A2 | 1.01 | 0.99-1.03 | 0.384 | NA | NA | NA |
| ULK3 | 1.02 | 1-1.03 | 0.021 | 1.03 | 1.02-1.05 | 0 |
| UNC45B | 0.15 | 0-16.94 | 0.43 | NA | NA | NA |
| USF1 | 1.01 | 1-1.01 | 0.001 | 0.97 | 0.97-0.98 | 0 |
| USP50 | 0.63 | 0.18-2.24 | 0.472 | NA | NA | NA |
| UTP3 | 1.03 | 1.01-1.04 | 0 | 1.25 | 1.23-1.27 | 0 |
| VAC14 | 1.02 | 0.99-1.04 | 0.147 | NA | NA | NA |
| VPS16 | 1.02 | 1.01-1.03 | 0.001 | 0.62 | 0.61-0.63 | 0 |
| VPS45 | 1.05 | 1.03-1.07 | 0 | 0.88 | 0.86-0.9 | 0 |
| VWA8 | 0.99 | 0.96-1.01 | 0.218 | NA | NA | NA |
| WDHD1 | 1.15 | 1.09-1.21 | 0 | 23.47 | 21.74-25.34 | 0 |
| WDR5B | 1.08 | 1.02-1.13 | 0.008 | 0.17 | 0.16-0.18 | 0 |
| WDR63 | 1.05 | 0.5-2.19 | 0.907 | NA | NA | NA |
| WDR81 | 1 | 0.98-1.02 | 0.849 | NA | NA | NA |
| WLS | 1 | 1-1.01 | 0.348 | NA | NA | NA |
| XPNPEP2 | 1 | 1.00-1.00 | 0.432 | NA | NA | NA |
| XRCC1 | 1.01 | 1-1.01 | 0.001 | 1.62 | 1.6-1.63 | 0 |
| YJEFN3 | 1.09 | 1.04-1.14 | 0 | 2 | 1.86-2.15 | 0 |
| YOD1 | 1.06 | 1.02-1.11 | 0.003 | 0.93 | 0.89-0.98 | 0.004 |
| YRDC | 1.03 | 1.01-1.04 | 0.008 | 0.85 | 0.83-0.87 | 0 |
| ZBED2 | 0.96 | 0.81-1.13 | 0.621 | NA | NA | NA |
| ZBED6CL | 1 | 1-1.01 | 0.646 | NA | NA | NA |
| ZBTB17 | 1.06 | 1.03-1.09 | 0 | 1.37 | 1.32-1.41 | 0 |
| ZBTB6 | 1.08 | 1.02-1.15 | 0.01 | 1.65 | 1.54-1.77 | 0 |
| ZCWPW1 | 1.01 | 0.95-1.07 | 0.76 | NA | NA | NA |
| ZDHHC23 | 1.03 | 0.99-1.07 | 0.096 | NA | NA | NA |
| ZDHHC8 | 1 | 0.98-1.03 | 0.851 | NA | NA | NA |
| ZFP41 | 1.07 | 1.01-1.12 | 0.016 | 1.17 | 1.09-1.24 | 0 |
| ZFP69B | 1.24 | 1.1-1.39 | 0.001 | 0.7 | 0.57-0.86 | 0.001 |
| ZKSCAN4 | 1.11 | 1.06-1.17 | 0 | 0.2 | 0.18-0.21 | 0 |
| ZMYM1 | 1.14 | 1.08-1.22 | 0 | 3.88 | 3.6-4.19 | 0 |
| ZNF134 | 1.03 | 0.99-1.07 | 0.129 | NA | NA | NA |
| ZNF174 | 1.11 | 1.05-1.17 | 0 | 4.12 | 3.89-4.37 | 0 |
| ZNF181 | 1.03 | 0.97-1.09 | 0.342 | NA | NA | NA |
| ZNF222 | 1.05 | 1.01-1.09 | 0.028 | 0.35 | 0.32-0.37 | 0 |
| ZNF227 | 1.04 | 1-1.08 | 0.061 | NA | NA | NA |
| ZNF275 | 1 | 0.97-1.02 | 0.856 | NA | NA | NA |
| ZNF282 | 1.02 | 1.01-1.04 | 0.001 | 2.02 | 1.99-2.06 | 0 |
| ZNF287 | 1.28 | 1.08-1.51 | 0.004 | 0.66 | 0.54-0.81 | 0 |
| ZNF302 | 1.02 | 1-1.04 | 0.063 | NA | NA | NA |
| ZNF304 | 1.06 | 1-1.12 | 0.056 | NA | NA | NA |
| ZNF334 | 1.06 | 0.94-1.19 | 0.377 | NA | NA | NA |
| ZNF395 | 1.02 | 1-1.04 | 0.012 | 0.75 | 0.73-0.76 | 0 |
| ZNF425 | 1.07 | 0.92-1.26 | 0.381 | NA | NA | NA |
| ZNF460 | 1.25 | 0.89-1.75 | 0.206 | NA | NA | NA |
| ZNF487 | 1.54 | 1.23-1.92 | 0 | 21.83 | 15.89-29.98 | 0 |
| ZNF501 | 1.03 | 0.88-1.2 | 0.728 | NA | NA | NA |
| ZNF507 | 1.05 | 1.01-1.09 | 0.027 | 1 | 0.94-1.06 | 0.889 |
| ZNF518A | 1.05 | 1.01-1.09 | 0.005 | 9.38 | 8.95-9.84 | 0 |
| ZNF518B | 1 | 0.87-1.14 | 0.988 | NA | NA | NA |
| ZNF565 | 1.21 | 1.01-1.45 | 0.04 | 0.03 | 0.02-0.03 | 0 |
| ZNF57 | 1.03 | 0.99-1.06 | 0.107 | NA | NA | NA |
| ZNF613 | 1.07 | 0.96-1.19 | 0.195 | NA | NA | NA |
| ZNF668 | 1.25 | 1.06-1.46 | 0.006 | 0.03 | 0.03-0.04 | 0 |
| ZNF736 | 1.12 | 1.04-1.21 | 0.004 | 0.64 | 0.56-0.74 | 0 |
| ZNF746 | 1.09 | 1.05-1.14 | 0 | 2.3 | 2.2-2.4 | 0 |
| ZNF763 | 1.13 | 0.94-1.35 | 0.188 | NA | NA | NA |
| ZNF777 | 1.04 | 1.01-1.06 | 0.002 | 0.72 | 0.7-0.74 | 0 |
| ZNF787 | 1.01 | 1-1.02 | 0.007 | 0.96 | 0.95-0.97 | 0 |
| ZNF805 | 1.04 | 0.85-1.28 | 0.685 | NA | NA | NA |
| ZNF880 | 1 | 0.96-1.04 | 0.999 | NA | NA | NA |
| ZSCAN9 | 1.11 | 1.07-1.16 | 0 | 14.53 | 13.84-15.25 | 0 |
| ZSWIM3 | 1.16 | 1.05-1.27 | 0.002 | 3.09 | 2.75-3.48 | 0 |
