# Supplementary material for: Identification of the Immune Subtype of Hepatocellular Carcinoma for the Prediction of Disease-Free Survival Time and Prevention of Recurrence by Integrated Analysis of Bulk- and Single-Cell RNA Sequencing Data
Source: Front Immunol. 2022 Jun 6;13:868325. doi: 10.3389/fimmu.2022.868325 (PMC9207181; doi:10.3389/fimmu.2022.868325)
Supplement: Supplementary file 10 [file Table_5.docx]

Table S5. Fifteen prognostic genes and their corresponding coefficients.

| Gene | Coefficients |
| --- | --- |
| AP000866.1 | 2.893454 |
| ATIC | 0.098522 |
| CAPN10 | -1.4446 |
| EDC3 | -0.02506 |
| EID3 | 0.86943 |
| NCKIPSD | 0.145852 |
| OXLD1 | 0.300641 |
| PHOSPHO2 | 2.290617 |
| POLE2 | 0.256851 |
| POLR3G | 0.190632 |
| SEPHS1 | 0.376175 |
| SRXN1 | 0.426397 |
| TIMM9 | 0.084766 |
| ZNF487 | 4.774708 |
| ZSCAN9 | 3.233664 |
