# Supplementary material for: Identification of the Immune Subtype of Hepatocellular Carcinoma for the Prediction of Disease-Free Survival Time and Prevention of Recurrence by Integrated Analysis of Bulk- and Single-Cell RNA Sequencing Data
Source: Front Immunol. 2022 Jun 6;13:868325. doi: 10.3389/fimmu.2022.868325 (PMC9207181; doi:10.3389/fimmu.2022.868325)
Supplement: Supplementary file 11 [file Table_6.docx]

Table S6. Results of correlation analyses between fifteen prognostic genes and drugs.

| Gene | Drug | Correlation coefficient | P value |
| --- | --- | --- | --- |
| SRXN1 | Imexon | -0.61238 | 2.00E-07 |
| SRXN1 | Arsenic trioxide | -0.58978 | 7.10E-07 |
| SRXN1 | 3-Bromopyruvate (acid) | -0.56195 | 2.98E-06 |
| SRXN1 | Chelerythrine | -0.52534 | 1.63E-05 |
| SRXN1 | Irofulven | 0.524767 | 1.68E-05 |
| SRXN1 | Nelarabine | -0.52455 | 1.69E-05 |
| SRXN1 | 7-Hydroxystaurosporine | -0.5242 | 1.72E-05 |
| SRXN1 | Carmustine | -0.49732 | 5.29E-05 |
| CAPN10 | Nelarabine | 0.483928 | 8.95E-05 |
| SRXN1 | Dimethylaminoparthenolide | -0.48217 | 9.58E-05 |
| CAPN10 | Asparaginase | 0.467876 | 0.000164 |
| SRXN1 | Cyclophosphamide | -0.46381 | 0.00019 |
| SRXN1 | Ifosfamide | -0.4609 | 0.000211 |
| SEPHS1 | Nelarabine | 0.457108 | 0.000241 |
| POLE2 | Chelerythrine | 0.446149 | 0.000353 |
| SEPHS1 | Chelerythrine | 0.443913 | 0.000381 |
| CAPN10 | Cladribine | 0.436698 | 0.000486 |
| SRXN1 | Fenretinide | -0.42981 | 0.00061 |
| ZNF487 | Mithramycin | -0.42084 | 0.000813 |
| SRXN1 | Bendamustine | -0.42023 | 0.000829 |
| EID3 | Irofulven | 0.419854 | 0.000839 |
| SRXN1 | Fluphenazine | -0.41831 | 0.000881 |
| ZNF487 | Depsipeptide | -0.4172 | 0.000912 |
| CAPN10 | Pipobroman | 0.416657 | 0.000928 |
| EID3 | Abiraterone | -0.41361 | 0.00102 |
| SRXN1 | Lomustine | -0.41285 | 0.001045 |
| SRXN1 | Parthenolide | -0.41281 | 0.001046 |
| TIMM9 | Chelerythrine | 0.41105 | 0.001104 |
| EID3 | Bortezomib | -0.41014 | 0.001136 |
| CAPN10 | Fludarabine | 0.410054 | 0.001139 |
| ATIC | Vorinostat | 0.404883 | 0.001333 |
| EID3 | Tamoxifen | -0.40134 | 0.001482 |
| SRXN1 | XK-469 | -0.39939 | 0.001571 |
| SRXN1 | Dacarbazine | -0.39527 | 0.001774 |
| ZNF487 | Hydrastinine HCl | -0.39379 | 0.001852 |
| SRXN1 | Hydroxyurea | -0.39342 | 0.001872 |
| POLR3G | Everolimus | -0.39285 | 0.001904 |
| SRXN1 | Fostamatinib | -0.39258 | 0.001919 |
| OXLD1 | Hydroxyurea | 0.391138 | 0.002001 |
| ZNF487 | Actinomycin D | -0.39055 | 0.002035 |
| EID3 | Raloxifene | -0.3882 | 0.002177 |
| CAPN10 | Chelerythrine | 0.387798 | 0.002203 |
| SRXN1 | 6-Mercaptopurine | -0.38653 | 0.002284 |
| SRXN1 | Melphalan | -0.38632 | 0.002297 |
| OXLD1 | Dasatinib | -0.3862 | 0.002305 |
| CAPN10 | Triethylenemelamine | 0.380719 | 0.002692 |
| CAPN10 | Thiotepa | 0.379515 | 0.002784 |
| SRXN1 | kahalide f | 0.378891 | 0.002833 |
| SRXN1 | Palbociclib | -0.37624 | 0.003049 |
| ZSCAN9 | Fluorouracil | -0.3741 | 0.003235 |
| EID3 | Bafetinib | -0.37318 | 0.003317 |
| OXLD1 | Cladribine | 0.371717 | 0.003452 |
| SRXN1 | Oxaliplatin | -0.37157 | 0.003466 |
| CAPN10 | Chlorambucil | 0.368791 | 0.003737 |
| SRXN1 | PX-316 | -0.36824 | 0.003793 |
| TIMM9 | Ifosfamide | 0.36792 | 0.003826 |
| SRXN1 | Chlorambucil | -0.36711 | 0.003911 |
| ZSCAN9 | Cordycepin | 0.366983 | 0.003924 |
| EID3 | SR16157 | -0.36607 | 0.004021 |
| SEPHS1 | Vemurafenib | -0.36595 | 0.004035 |
| OXLD1 | Triethylenemelamine | 0.365899 | 0.00404 |
| ATIC | Nelarabine | 0.365152 | 0.004121 |
| OXLD1 | Uracil mustard | 0.363353 | 0.004323 |
| ZNF487 | geldanamycin analog | -0.36196 | 0.004486 |
| ATIC | AT-13387 | 0.360055 | 0.004717 |
| ATIC | Chelerythrine | 0.358991 | 0.004851 |
| ZNF487 | Homoharringtonine | -0.35732 | 0.005067 |
| ZNF487 | Clofarabine | 0.356654 | 0.005156 |
| CAPN10 | Uracil mustard | 0.35649 | 0.005178 |
| POLR3G | Bafetinib | 0.352291 | 0.005771 |
| SRXN1 | Raloxifene | -0.35137 | 0.005909 |
| CAPN10 | Cytarabine | 0.351184 | 0.005937 |
| SRXN1 | Obatoclax | -0.35096 | 0.00597 |
| OXLD1 | Pipobroman | 0.35083 | 0.005991 |
| ZNF487 | Pipamperone | -0.34973 | 0.006161 |
| CAPN10 | Clofarabine | 0.34969 | 0.006167 |
| NCKIPSD | Fludarabine | 0.349488 | 0.006199 |
| OXLD1 | tfdu | 0.348447 | 0.006365 |
| CAPN10 | Decitabine | 0.348368 | 0.006378 |
| PHOSPHO2 | Irofulven | -0.34416 | 0.007091 |
| CAPN10 | Dexamethasone Decadron | 0.343976 | 0.007123 |
| POLR3G | bisacodyl, active ingredient of viraplex | -0.34294 | 0.007311 |
| ZNF487 | Eribulin mesilate | -0.34247 | 0.007395 |
| POLR3G | Acetalax | -0.34222 | 0.007442 |
| POLE2 | Nelarabine | 0.34184 | 0.007512 |
| ZSCAN9 | Actinomycin D | -0.34092 | 0.007685 |
| SEPHS1 | Asparaginase | 0.340168 | 0.00783 |
| SRXN1 | Alectinib | -0.33952 | 0.007956 |
| POLR3G | Vemurafenib | 0.337233 | 0.008416 |
| EID3 | Hypothemycin | -0.33579 | 0.008718 |
| POLE2 | O-6-Benzylguanine | -0.33536 | 0.00881 |
| OXLD1 | Chlorambucil | 0.33523 | 0.008837 |
| SEPHS1 | 8-Chloro-adenosine | 0.334805 | 0.008929 |
| EID3 | Dabrafenib | -0.33422 | 0.009057 |
| CAPN10 | Carboplatin | 0.333364 | 0.009246 |
| ZSCAN9 | Megestrol acetate | 0.332882 | 0.009354 |
| OXLD1 | Vorinostat | 0.332156 | 0.009519 |
| EID3 | Isotretinoin | -0.3321 | 0.009533 |
| CAPN10 | 5-fluoro deoxy uridine 10mer | 0.332003 | 0.009554 |
| OXLD1 | Thiotepa | 0.330849 | 0.009823 |
| CAPN10 | Fluphenazine | 0.329844 | 0.010062 |
| SRXN1 | Uracil mustard | -0.32878 | 0.01032 |
| SEPHS1 | Dabrafenib | -0.32696 | 0.010776 |
| EID3 | Vemurafenib | -0.3266 | 0.010869 |
| EID3 | Digoxin | 0.326429 | 0.010913 |
| ATIC | Cladribine | 0.325969 | 0.011032 |
| EDC3 | Vorinostat | 0.325934 | 0.011042 |
| CAPN10 | Gemcitabine | 0.323588 | 0.011668 |
| SRXN1 | Dexamethasone Decadron | -0.32355 | 0.01168 |
| CAPN10 | XK-469 | 0.323313 | 0.011743 |
| ATIC | Olaparib | -0.32319 | 0.011777 |
| OXLD1 | LMP-400 | 0.322514 | 0.011965 |
| SEPHS1 | Chlorambucil | 0.322385 | 0.012001 |
| OXLD1 | Etoposide | 0.322238 | 0.012042 |
| SEPHS1 | Cladribine | 0.322038 | 0.012098 |
| OXLD1 | Gemcitabine | 0.321668 | 0.012203 |
| PHOSPHO2 | Seliciclib | -0.32131 | 0.012304 |
| OXLD1 | 5-fluoro deoxy uridine 10mer | 0.320928 | 0.012415 |
| OXLD1 | Irinotecan | 0.320708 | 0.012479 |
| OXLD1 | Cytarabine | 0.320478 | 0.012545 |
| NCKIPSD | AFP464 | -0.3203 | 0.012597 |
| SEPHS1 | Fludarabine | 0.319998 | 0.012686 |
| POLE2 | Amonafide | 0.319119 | 0.012946 |
| CAPN10 | Cisplatin | 0.317593 | 0.01341 |
| CAPN10 | Hydroxyurea | 0.317534 | 0.013428 |
| TIMM9 | 3-Bromopyruvate (acid) | 0.317388 | 0.013473 |
| OXLD1 | Teniposide | 0.315175 | 0.014173 |
| POLR3G | Erlotinib | -0.31419 | 0.014494 |
| OXLD1 | Asparaginase | 0.313976 | 0.014565 |
| OXLD1 | Entinostat | 0.312652 | 0.015009 |
| ATIC | Everolimus | -0.31255 | 0.015043 |
| ZNF487 | BN-2629 | -0.30981 | 0.016 |
| ZNF487 | okadaic acid | -0.30968 | 0.016048 |
| ATIC | Parthenolide | 0.309397 | 0.01615 |
| ZSCAN9 | Mithramycin | -0.30756 | 0.016827 |
| EID3 | Crizotinib | -0.30711 | 0.016994 |
| POLE2 | Zoledronate | -0.30653 | 0.017215 |
| PHOSPHO2 | Allopurinol | 0.305941 | 0.017442 |
| ZNF487 | Doxorubicin | -0.30537 | 0.017662 |
| CAPN10 | Dasatinib | -0.30462 | 0.017956 |
| OXLD1 | Idarubicin | 0.3046 | 0.017966 |
| PHOSPHO2 | Fluphenazine | 0.30455 | 0.017986 |
| POLE2 | Rapamycin | -0.30425 | 0.018104 |
| TIMM9 | Amonafide | 0.304052 | 0.018184 |
| POLR3G | Vandetanib | -0.30283 | 0.018678 |
| EID3 | Ixazomib citrate | -0.30282 | 0.018682 |
| EID3 | Imexon | -0.3025 | 0.018812 |
| PHOSPHO2 | Bleomycin | -0.30238 | 0.018863 |
| CAPN10 | Digoxin | 0.302297 | 0.018898 |
| ZNF487 | Dolastatin 10 | -0.30188 | 0.019071 |
| SEPHS1 | Allopurinol | 0.30159 | 0.019192 |
| ZNF487 | ABT-199 | -0.30092 | 0.019475 |
| ATIC | Trametinib | 0.299654 | 0.020018 |
| NCKIPSD | Palbociclib | -0.29848 | 0.020532 |
| POLE2 | Everolimus | -0.29839 | 0.020572 |
| SRXN1 | Asparaginase | -0.29719 | 0.021112 |
| OXLD1 | Fulvestrant | 0.296924 | 0.021234 |
| OXLD1 | Nitrogen mustard | 0.296911 | 0.021239 |
| ATIC | Asparaginase | 0.296098 | 0.021614 |
| POLE2 | Temsirolimus | -0.29529 | 0.02199 |
| ATIC | LY-294002 | -0.29476 | 0.022243 |
| PHOSPHO2 | Nelarabine | 0.294751 | 0.022245 |
| ZSCAN9 | Depsipeptide | -0.29452 | 0.022354 |
| SEPHS1 | Denileukin Diftitox Ontak | -0.29444 | 0.022393 |
| ATIC | Abiraterone | -0.29431 | 0.022455 |
| SEPHS1 | okadaic acid | -0.29303 | 0.023076 |
| TIMM9 | Hydroxyurea | 0.292927 | 0.023126 |
| EDC3 | XL-147 | 0.292733 | 0.023221 |
| OXLD1 | Oxaliplatin | 0.292503 | 0.023335 |
| OXLD1 | LMP776 | 0.292077 | 0.023546 |
| CAPN10 | XL-147 | 0.290988 | 0.024094 |
| SEPHS1 | Uracil mustard | 0.290911 | 0.024133 |
| PHOSPHO2 | Everolimus | -0.29086 | 0.024157 |
| OXLD1 | Dromostanolone Propionate | 0.290358 | 0.024416 |
| CAPN10 | Melphalan | 0.289923 | 0.024641 |
| POLE2 | 6-Mercaptopurine | 0.28988 | 0.024663 |
| SEPHS1 | Fenretinide | 0.289462 | 0.02488 |
| ZSCAN9 | Doxorubicin | -0.28938 | 0.024923 |
| POLE2 | Hydroxyurea | 0.288999 | 0.025123 |
| PHOSPHO2 | Mitomycin | -0.28869 | 0.025287 |
| EDC3 | Karenitecin | 0.288271 | 0.025509 |
| SRXN1 | Imatinib | -0.28816 | 0.025565 |
| EDC3 | 5-fluoro deoxy uridine 10mer | 0.28776 | 0.025782 |
| EID3 | Carmustine | -0.28761 | 0.02586 |
| ZNF487 | Imexon | -0.28743 | 0.025959 |
| ATIC | Cytarabine | 0.28657 | 0.026429 |
| SEPHS1 | Hydroxyurea | 0.285535 | 0.027003 |
| SRXN1 | Perifosine | -0.28536 | 0.027101 |
| NCKIPSD | Cladribine | 0.285359 | 0.027101 |
| CAPN10 | Elliptinium Acetate | 0.285253 | 0.027161 |
| TIMM9 | Vemurafenib | -0.28525 | 0.027164 |
| EID3 | LDK-378 | -0.28522 | 0.027178 |
| CAPN10 | Raltitrexed | 0.285196 | 0.027193 |
| PHOSPHO2 | Ponatinib | 0.285178 | 0.027203 |
| EDC3 | Dasatinib | -0.28444 | 0.027622 |
| EDC3 | Midostaurin | -0.28255 | 0.028717 |
| TIMM9 | Imexon | 0.282416 | 0.028795 |
| ZNF487 | Daunorubicin | -0.2818 | 0.029159 |
| CAPN10 | Aminoflavone | -0.28128 | 0.029469 |
| PHOSPHO2 | Celecoxib | 0.281025 | 0.029626 |
| POLE2 | Belinostat | 0.280796 | 0.029764 |
| SRXN1 | Trametinib | 0.280729 | 0.029805 |
| ZNF487 | Dimethylaminoparthenolide | -0.28051 | 0.029936 |
| POLE2 | Ifosfamide | 0.280497 | 0.029946 |
| NCKIPSD | Depsipeptide | -0.28013 | 0.03017 |
| ATIC | Cobimetinib (isomer 1) | 0.278695 | 0.031062 |
| EID3 | Fludarabine | 0.278552 | 0.031152 |
| ATIC | Methotrexate | 0.277081 | 0.032091 |
| CAPN10 | Irinotecan | 0.276528 | 0.03245 |
| POLR3G | Dabrafenib | 0.275916 | 0.032852 |
| EDC3 | Nelarabine | 0.275902 | 0.032861 |
| ZNF487 | Carfilzomib | -0.27513 | 0.033374 |
| OXLD1 | Clofarabine | 0.274169 | 0.03402 |
| SEPHS1 | Pyrazoloacridine | 0.273886 | 0.034213 |
| TIMM9 | Everolimus | -0.27355 | 0.034444 |
| POLE2 | Itraconazole | -0.27323 | 0.03466 |
| ZNF487 | Epirubicin | -0.2731 | 0.034755 |
| ATIC | Hydroxyurea | 0.272318 | 0.035295 |
| NCKIPSD | Vinorelbine | -0.27212 | 0.035433 |
| SRXN1 | BN-2629 | -0.27187 | 0.035611 |
| POLR3G | Afatinib | -0.27182 | 0.035643 |
| ZNF487 | Tanespimycin | -0.27182 | 0.035648 |
| SRXN1 | Vorinostat | -0.27152 | 0.035854 |
| OXLD1 | XK-469 | 0.270291 | 0.036737 |
| POLR3G | Cobimetinib (isomer 1) | 0.270067 | 0.0369 |
| ATIC | Fludarabine | 0.269982 | 0.036962 |
| POLE2 | By-Product of CUDC-305 | 0.269535 | 0.037288 |
| SEPHS1 | Clofarabine | 0.269134 | 0.037582 |
| CAPN10 | LMP-400 | 0.268988 | 0.03769 |
| OXLD1 | Mitomycin | 0.268825 | 0.03781 |
| EID3 | Selumetinib | -0.26853 | 0.03803 |
| TIMM9 | Palbociclib | 0.268146 | 0.038316 |
| POLE2 | Fenretinide | 0.268021 | 0.03841 |
| POLR3G | O-6-Benzylguanine | -0.2676 | 0.038729 |
| CAPN10 | Idarubicin | 0.267393 | 0.038883 |
| SRXN1 | Denileukin Diftitox Ontak | -0.26738 | 0.038892 |
| PHOSPHO2 | LY-294002 | -0.26728 | 0.038966 |
| SEPHS1 | 6-Mercaptopurine | 0.267093 | 0.039111 |
| TIMM9 | Fluorouracil | -0.26699 | 0.039193 |
| PHOSPHO2 | 5-fluoro deoxy uridine 10mer | -0.26691 | 0.03925 |
| NCKIPSD | Celecoxib | -0.26621 | 0.039792 |
| ZSCAN9 | Bendamustine | -0.26606 | 0.039903 |
| SEPHS1 | Parthenolide | 0.265235 | 0.040548 |
| SRXN1 | Nitrogen mustard | -0.2644 | 0.041204 |
| EID3 | Pipamperone | -0.2643 | 0.041291 |
| SRXN1 | Pipamperone | -0.26412 | 0.041428 |
| ZNF487 | Pyrazoloacridine | 0.264008 | 0.04152 |
| ATIC | Itraconazole | -0.264 | 0.041528 |
| ZNF487 | Vinblastine | -0.26377 | 0.04171 |
| PHOSPHO2 | Imexon | 0.263556 | 0.041883 |
| NCKIPSD | AP-26113 | -0.2634 | 0.042008 |
| POLR3G | Hypothemycin | 0.262964 | 0.042362 |
| EID3 | Denileukin Diftitox Ontak | -0.26258 | 0.042678 |
| CAPN10 | Batracylin | 0.262431 | 0.042798 |
| TIMM9 | PX-316 | 0.262032 | 0.043127 |
| TIMM9 | Nelarabine | 0.261935 | 0.043207 |
| SRXN1 | Isotretinoin | -0.2614 | 0.043647 |
| EID3 | 7-Hydroxystaurosporine | -0.26118 | 0.043833 |
| POLR3G | Selumetinib | 0.260428 | 0.044468 |
| CAPN10 | LY-294002 | -0.2599 | 0.044917 |
| ZNF487 | Alvespimycin | -0.25982 | 0.044989 |
| ATIC | Amonafide | 0.259567 | 0.045201 |
| SRXN1 | Carboplatin | -0.25954 | 0.045225 |
| SRXN1 | Calusterone | -0.25947 | 0.045287 |
| EID3 | 5-fluoro deoxy uridine 10mer | 0.257782 | 0.046755 |
| NCKIPSD | 5-fluoro deoxy uridine 10mer | 0.257745 | 0.046788 |
| OXLD1 | Staurosporine | -0.25744 | 0.047054 |
| OXLD1 | Valrubicin | 0.25743 | 0.047066 |
| ZSCAN9 | Lapachone | 0.257185 | 0.047284 |
| SEPHS1 | PX-316 | 0.256776 | 0.047649 |
| OXLD1 | Dexrazoxane | 0.256695 | 0.047722 |
| SRXN1 | Sonidegib | 0.256342 | 0.048039 |
| OXLD1 | Topotecan | 0.255842 | 0.048492 |
| SEPHS1 | Amonafide | 0.255241 | 0.049042 |
| SEPHS1 | 3-Bromopyruvate (acid) | 0.254877 | 0.049376 |
| ATIC | Rapamycin | -0.25426 | 0.049945 |
